# A conserved role for AMP-activated protein kinase in NGLY1 deficiency

**DOI:** 10.1371/journal.pgen.1009258

**Published:** 2020-12-14

**Authors:** Seung Yeop Han, Ashutosh Pandey, Tereza Moore, Antonio Galeone, Lita Duraine, Tina M. Cowan, Hamed Jafar-Nejad

**Affiliations:** 1 Department of Molecular and Human Genetics, Baylor College of Medicine, Houston, Texas, United States of America; 2 Department of Pathology, Stanford University, Stanford, California, United States of America; 3 Howard Hughes Medical Institute, Baylor College of Medicine, Houston, Texas, United States of America; 4 Jan & Dan Duncan Neurological Research Institute Center, Texas Children’s Hospital, Houston, Texas, United States of America; 5 Genetics & Genomics Graduate Program, Baylor College of Medicine, Houston, Texas, United States of America; 6 Development, Disease Models & Therapeutics Graduate Program, Baylor College of Medicine, Houston, Texas, United States of America; 7 Program in Developmental Biology, Baylor College of Medicine, Houston, Texas, United States of America; Department of Biosciences & Institute of Biotechnology, FINLAND

## Abstract

Mutations in human *N*-glycanase 1 (*NGLY1*) cause the first known congenital disorder of deglycosylation (CDDG). Patients with this rare disease, which is also known as *NGLY1* deficiency, exhibit global developmental delay and other phenotypes including neuropathy, movement disorder, and constipation. NGLY1 is known to regulate proteasomal and mitophagy gene expression through activation of a transcription factor called "nuclear factor erythroid 2-like 1" (NFE2L1). Loss of NGLY1 has also been shown to impair energy metabolism, but the molecular basis for this phenotype and its *in vivo* consequences are not well understood. Using a combination of genetic studies, imaging, and biochemical assays, here we report that loss of NGLY1 in the visceral muscle of the *Drosophila* larval intestine results in a severe reduction in the level of AMP-activated protein kinase α (AMPKα), leading to energy metabolism defects, impaired gut peristalsis, failure to empty the gut, and animal lethality. *Ngly1*^*–/–*^ mouse embryonic fibroblasts and *NGLY1* deficiency patient fibroblasts also show reduced *AMPKα* levels. Moreover, pharmacological activation of AMPK signaling significantly suppressed the energy metabolism defects in these cells. Importantly, the reduced AMPKα level and impaired energy metabolism observed in *NGLY1* deficiency models are not caused by the loss of NFE2L1 activity. Taken together, these observations identify reduced AMPK signaling as a conserved mediator of energy metabolism defects in *NGLY1* deficiency and suggest AMPK signaling as a therapeutic target in this disease.

## Author summary

Thousands of proteins in most organisms harbor a type of sugar modification called *N*-glycan. Addition of *N*-glycans to proteins has long been known to affect protein quality control and function. However, recent studies have shown that removing *N*-glycans from proteins also plays key roles in cellular biology and animal development. Importantly, children with mutations in the enzyme responsible for *N*-glycan removal (NGLY1) exhibit developmental delay, seizures, movement disorder, and other abnormalities. NGLY1 is responsible for adjusting the activity of proteasome, a cellular machinery involved in protein degradation. NGLY1 has also been linked to the function and homeostasis of mitochondria, the major energy production engine in animal cells. However, the link between these processes and patient symptoms is not clear, and no therapies exist for this disease. We now report that loss of NGLY1 results in reduced levels of a cellular energy sensor called AMPKα in fruit flies, mouse cells and patient cells. Restoring AMPKα level or its pharmacological activation improves the energy homeostasis defects in all three systems and significantly increases the survival of NGLY1-mutant fruit flies independently of proteasome. Our data suggest that enhancement of AMPKα activity can serve as a potential therapeutic approach in NGLY1 deficiency patients.

## Introduction

The cytoplasmic enzyme *N*-glycanase 1 (NGLY1) catalyzes the removal of *N*-linked glycans from glycoproteins and is thought to operate as part of the endoplasmic reticulum-associated degradation (ERAD) pathway [1]. Recessive mutations in human *NGLY1* result in a genetic disorder with various phenotypes including developmental delay, seizures, hypo-/alacrima, elevated liver enzymes, diminished deep tendon reflexes, muscle weakness, orthopedic manifestations, and chronic constipation [2–8]. This disease is a congenital disorder of deglycosylation (OMIM # 615273) and is commonly referred to as *NGLY1* deficiency. NGLY1 and its homologs in model organisms have been shown to regulate the proteasomal gene expression by deglycosylating a transcription factor called "nuclear factor erythroid 2-like 1" (NFE2L1; also called NRF1; SKN-1 in worms) [9–11]. However, the extent to which this proteasomal defect contributes to *NGLY1* deficiency phenotypes in human patients and developmental abnormalities observed in *Ngly1*-mutant animal models remains to be determined. It is worth mentioning that NFE2L1 and its paralog NFE2L2 have also been called NRF1 and NRF2. However, since NRF1 is the official symbol for a distinct protein called nuclear respiratory factor 1 in mammals, we will use NFE2L1 and NFE2L2 in the current work.

Studies in tissue samples from *NGLY1* deficiency patients, patient fibroblasts, *Ngly1*^*–/–*^ mouse embryonic fibroblasts (MEFs) and *C*. *elegans* mutants for the *NGLY1* homolog (*png-1*^*–/–*^) have shown structural and functional abnormalities in mitochondria [7,12]. Moreover, *Ngly1*^*–/–*^ MEFs fail to properly clear damaged mitochondria via mitophagy [13]. Given that a number of *NGLY1* deficiency phenotypes like developmental delay, neuropathy, muscle weakness, and seizures are also observed in mitochondrial disorders [14], they are considered to be one of the differential diagnoses in patients suspected of having *NGLY1* deficiency [2]. Therefore, although a specific phenotype in human *NGLY1* deficiency patients and *Ngly1*-mutant animals is yet to be directly linked to energy homeostasis defects, these studies suggest that mitochondrial abnormalities might contribute to some aspects of *NGLY1* deficiency pathophysiology, and that improving energy homeostasis might be beneficial in this patient population.

The *Drosophila* genome encodes a single NGLY1 homolog called PNGase-like or Pngl [15]. Under regular culture conditions, *Pngl*-null (*Pngl*^*–/–*^) *Drosophila* show a significant developmental delay and ~99% lethality [15,16]. We have previously reported that 80%-85% of the lethality observed in *Pngl*^*–/–*^ animals can be rescued by transgenic expression of human NGLY1 in the mesoderm [16]. Around 20–30% of the lethality can be explained by a tissue-specific requirement for Pngl in the visceral mesoderm to promote bone morphogenetic protein (BMP) signaling mediated by the fly BMP protein Decapentaplegic or Dpp [16]. *Pngl*^*–/–*^ larvae also show a food accumulation phenotype (severe failure in gut clearance) that cannot be explained by the loss of BMP signaling [16]. However, the molecular basis of gut clearance defects and their contribution to the lethality observed in *Pngl*^*–/–*^ animals remained to be identified. Moreover, it was not clear whether at a mechanistic level the regulation of *Drosophila* gut clearance by Pngl has any parallels in mammals.

Here, we show that the food accumulation phenotype in *Pngl*^*–/–*^ larvae is caused by reduced mesodermal expression of *AMP-activated protein kinase α subunit* (*AMPKα*), which encodes the catalytic subunit for a major energy sensor in the cells [17]. The midgut in *Pngl*^*–/–*^ larvae exhibits abnormal mitochondrial cristae, reduced ATP content and increased oxidative stress, all of which can be improved upon restoring *AMPKα* levels in the mesoderm. Importantly, both *Ngly1*^*–/–*^ MEFs and fibroblasts from *NGLY1* deficiency patients show a similar reduction in *AMPKα1* and *AMPKα2* expression. Moreover, pharmacological enhancement of AMPK signaling rescues the impaired energy homeostasis in both model systems. Importantly, the reduction in *AMPKα* level cannot be explained by impaired proteasomal gene expression in *NGLY1*-deficient models. Our work identifies reduced *AMPKα* expression as an evolutionarily conserved mechanism contributing to the energy homeostasis defects in *NGLY1*-deficient animals and suggests AMPK signaling as a potential therapeutic target in *NGLY1* deficiency patients.

## Results

### *Drosophila Pngl* is required in the mesoderm but not neurons or endoderm to promote midgut peristalsis

**Fig 1:**
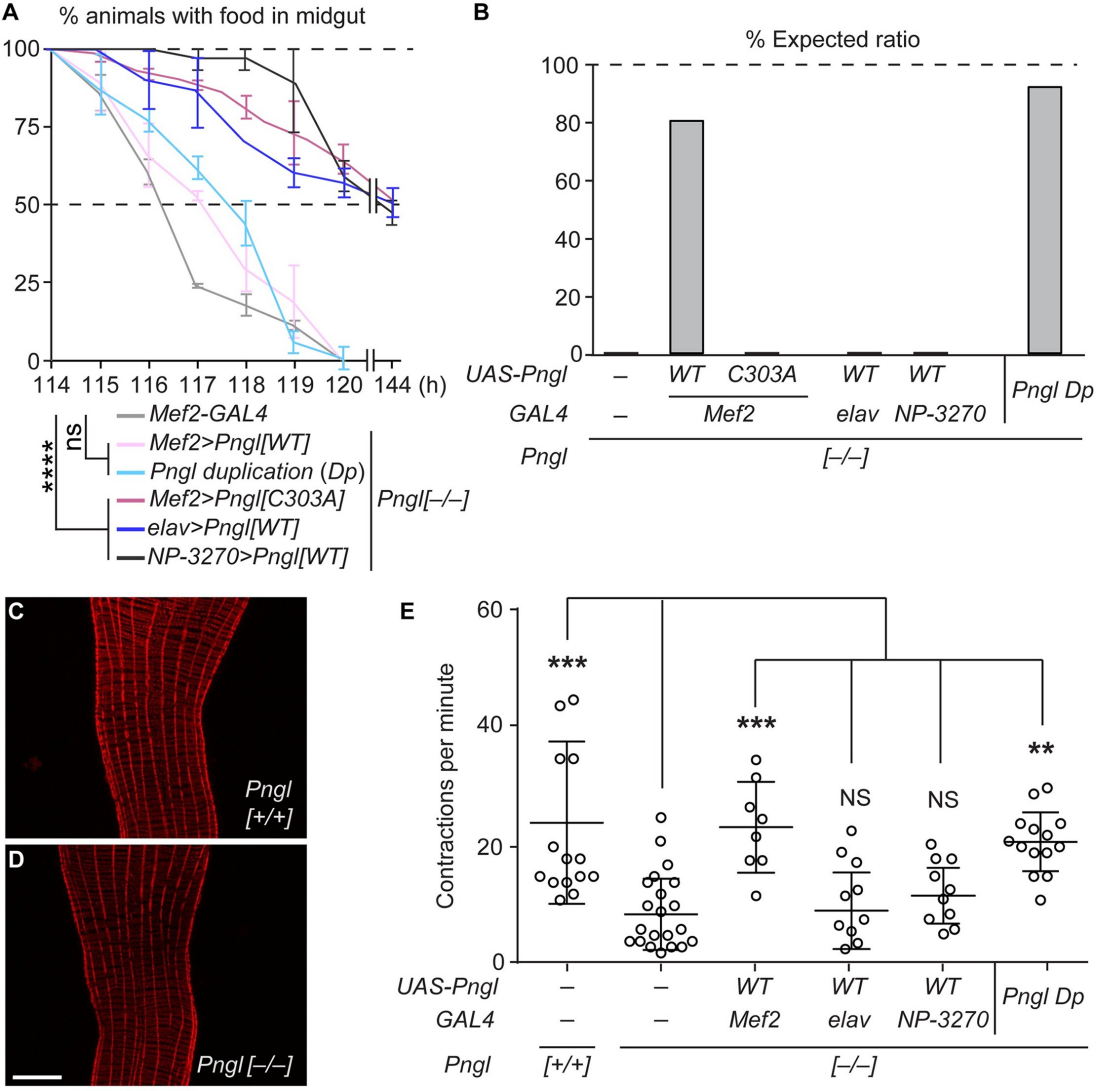
The food accumulation phenotype in *Pngl* mutants is associated with gut peristalsis defects and is rescued by mesoderm-specific enzymatic activity of Pngl. (A,B) Gut clearance assays in 3rd instar larvae and lethality rescue assays of the indicated genotypes are shown. The x-axis shows hours (h) after egg laying. The food accumulation phenotype (A) and the lethality (B) of *Pngl* mutants is rescued by a *Pngl* genomic duplication (*Pngl Dp*) and by overexpressing wild-type (*WT)* Pngl by a mesodermal driver (*Mef2-GAL4*) but not by expressing an enzymatic-deficient *Pngl* mutant (*C303A*) in the mesoderm or by expressing *Pngl*^*WT*^ using neuronal (*elav-GAL4*) and endodermal (*NP-3270-GAL4*) drivers. Data in (A) represent mean ± SD of three independent experiments. Animal numbers in (B) are 200, 240, 160, 130, 193, and 145 from left to right. (C,D) Representative images of midguts of *y w* control (C) and *Pngl*^*–/–*^ (D) larvae stained with phalloidin (red) do not show gross morphological defects in the mutant visceral muscle. The scale bar in D is 50 μm and applies to (C) as well. (E) Midgut contraction frequencies per minute in *Pngl*^*–/–*^ larvae is significantly reduced compared to control animals and is rescued by *Pngl Dp* and mesodermal but not neuronal or endodermal overexpression of *Pngl*^*WT*^. Each circle represents a larva. Significance is ascribed as **P<0.01 and ***P<0.001. NS, not significant.

To determine the mechanism for the food accumulation phenotypes observed in *Pngl* mutant larvae, we first performed transgenic rescue experiments. Overexpression of wild-type *Pngl* in the mesoderm by using the *GAL4/UAS* system [18] fully rescued the food accumulation phenotype of *Pngl*^*–/–*^ larvae, similar to adding one copy a *Pngl* genomic transgene, *Pngl Dp* (Fig 1A and S1 Fig). Moreover, mesodermal overexpression (*Mef2-GAL4*) of *Pngl* with a C303A mutation in its catalytic domain failed to rescue the food accumulation phenotype in *Pngl*^*–/–*^ larvae (Fig 1A). Similar to human NGLY1, mesodermal expression of the fly *Pngl* (but not *Pngl*^*C303A*^) rescued the lethality in ~80% of *Pngl*^*–/–*^ animals (Fig 1B). However, none of these phenotypes were rescued by endodermal (*NP-3270-GAL4*) or neuronal (*elav-GAL4*) overexpression of *Pngl* in *Pngl*^*–/–*^ animals (Fig 1A and 1B). Together, these observations indicate a critical role for Pngl’s enzymatic activity in the mesoderm to ensure gut clearance and animal survival.

Phalloidin staining and transmission electron microscopy (TEM) of the visceral muscles in third instar larvae do not show an obvious morphological defect in *Pngl*^*–/–*^ animals (Fig 1C and 1D and S2 Fig). To examine whether the food accumulation phenotype of *Pngl*^*–/–*^ larvae can be explained by a functional defect in the midgut, we assessed the effect of loss of *Pngl* on the peristaltic activity in freshly dissected larval midguts. We found a significant decrease in the number of midgut contractions per minute in *Pngl*^*–/–*^ midguts compared to control midguts (Fig 1E). This phenotype was rescued by introducing genomic *Pngl Dp* or by overexpressing *Pngl* in the mesoderm, but not in neurons or endoderm (Fig 1E). These observations indicate that *Pngl* is required in the visceral muscle to promote midgut peristalsis and gut clearance.

### *AMPKα* expression is reduced in *Pngl*^*–/–*^ larval midguts

**Fig 2:**
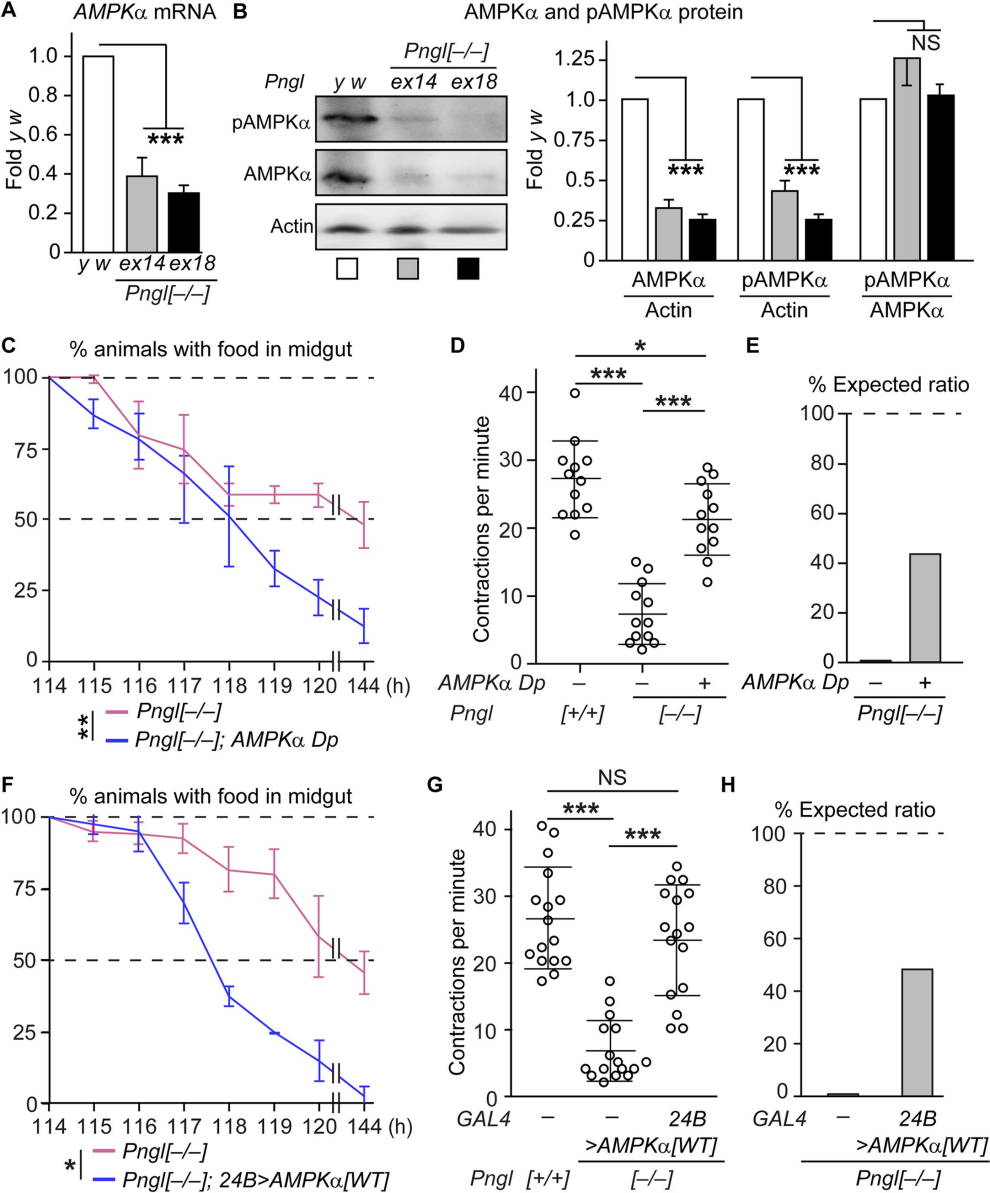
Restoration of *AMPKα* levels suppresses food accumulation phenotype, gut peristalsis defects, and lethality in *Pngl* mutants. (A) qRT-PCR assays show a significant decrease in the expression level of *AMPKα* mRNA in the midguts of *Pngl* mutant larvae (*Pngl*^*ex14/ex14*^ and *Pngl*^*ex18/ex18*^). (B) Western blotting and quantification of phospho-AMPKα (pAMPKα) and AMPKα expression in larval midguts show reduced expression in *Pngl* mutants. Note that the pAMPKα/AMPKα ratio is comparable in all three genotypes. (C-E) Adding a genomic *AMPKα* duplication (*AMPKα Dp*) in *Pngl*^*–/–*^ animals improved their gut clearance (C) and midgut contraction defect (D), and rescued their lethality by ~44% (E). (F-H) *AMPKα* overexpression in *Pngl*^*–/–*^ animals using a mesodermal driver (*how*^*24B*^*-GAL4*) improved their gut clearance (F), rescued their midgut contraction defect (G) and rescued their lethality by ~46% (H). The x-axis in C and F shows hours (h) after egg laying. Data in A-C and F represents mean ± SD of three independent experiments. Each circle in D and G represents a larva. Animal numbers for each genotype from left to right are 200 and 301 in (E), and 200 and 321 in (H). Significance is ascribed as *P<0.05, **P<0.01 and ***P<0.001. NS, not significant.

We previously reported that the food accumulation phenotype observed in *Pngl*^*–/–*^ larvae can be recapitulated by *Pngl* knock-down in the mesoderm by *how*^*24B*^*-GAL4*, but not by *dpp* knock-down using the same driver [16]. We recently reported that one copy of the *dpp*^*HA-3NQ*^ knock-in allele, in which three out of the four Dpp *N*-glycosylation sites are mutated, can fully rescue the BMP signaling defects in *Pngl*^*–/–*^ midguts [19]. However, this allele failed to improve the food accumulation phenotype in *Pngl*^*–/–*^ larvae, further indicating that the gut clearance phenotype in *Pngl* mutants is not caused by impaired BMP signaling (S3A Fig). A previous report had shown that *AMPKα* is required in the visceral muscle to promote peristaltic activity and gut clearance in *Drosophila* larvae [20]. Accordingly, we examined whether loss of *Pngl* affects *AMPKα* level and activation. As shown in Fig 2A, *AMPKα* mRNA expression level is significantly reduced in larval midguts homozygous for two different *Pngl* null alleles (*Pngl*^*ex14*^ and *Pngl*^*ex18*^; [15,16]) compared to the control *y w* animals. Western blotting showed a severe decrease in AMPKα and phospho-AMPKα (pAMPKα; active form) protein levels in *Pngl*^*ex14/ex14*^ and *Pngl*^*ex18/ex18*^ larval midguts compared to control midguts without changing the pAMPKα/AMPKα ratio (Fig 2B). These phenotypes can be fully rescued upon mesodermal overexpression of wild-type *Pngl* but not *Pngl*^*C303A*^, indicating that the Pngl enzymatic activity is required for the regulation of *AMPKα* level (S4A and S4B Fig). AMPKα functions as a heterotrimer with AMPKβ and AMPKγ [17]. However, loss of *Pngl* does not result in a statistically significant alteration in *AMPKβ* and *AMPKγ* mRNA levels in larval midguts (S4C Fig), suggesting that the observed effect is specific for the *AMPKα* subunit. These data suggest that Pngl is required for AMPKα expression in the fly midgut. Given the similar phenotypes observed here and previously [15,16] for these two null *Pngl* alleles, we have used *Pngl*^*ex14/ex14*^ animals in the rest of the current study and will refer to them as *Pngl*^*–/–*^.

### Restoration of *AMPK*α expression levels in the mesoderm suppresses the food accumulation and gut peristalsis phenotypes and partially rescues the lethality in *Pngl* mutant

If a decrease in midgut expression of *AMPKα* contributes to the *Pngl*^*–/–*^ food accumulation phenotype, increasing *AMPKα* expression should improve midgut emptying in these animals. To test this notion, we first used the genomic duplication *Dp(1;3)DC102*, *PBac{DC102}VK33* (referred to as *AMPKα Dp* hereafter), which contains *AMPKα* and seven neighboring genes (S4D Fig). One copy of this duplication increased *AMPKα* mRNA and AMPKα and pAMPKα protein levels in *Pngl*^*–/–*^ midguts (S4E and S4F Fig). This duplication also improved the gut clearance and significantly increased the number of midgut contractions per minute in *Pngl*^*–/–*^ animals (Fig 2C and 2D). Remarkably, adding a copy of this duplication increased the survival of *Pngl*^*–/–*^ animals to adulthood from ~1% to around 44% the expected Mendelian ratio (Fig 2E). As expected, the *AMPKα Dp* did not rescue the BMP-dependent phenotypes in *Pngl*^*–/–*^ midguts (S3B Fig). These observations strongly suggest that a reduction in *AMPKα* expression level underlies the gut clearance phenotypes of *Pngl*^*–/–*^ larvae and contributes to the lethality of these animals independently of the BMP signaling defects in these animals.

The severe reduction in AMPKα level in *Pngl*^*–/–*^ midguts (Fig 2A and 2B) and the previous report on the role of AMPKα expressed in the visceral muscle in promoting midgut peristalsis [20] together suggest that increasing the level of *AMPKα* in the visceral muscle by *AMPKα Dp* is sufficient for the rescue of both *Pngl*^*–/–*^ gut clearance and lethality. However, since this duplication harbors seven of *AMPKα*'s neighboring genes and because it should increase *AMPKα* expression in all cell types, we also performed tissue-specific rescue experiments. Overexpression of wild-type *AMPKα* (*AMPKα*^*WT*^) in the mesoderm by *how*^*24B*^*-GAL4* restored gut clearance and peristalsis activity in *Pngl*^*–/–*^ larvae (Fig 2F and 2G). Moreover, almost 46% of *Pngl*^*–/–*^ animals reached adulthood upon overexpression of *AMPKα*^*WT*^ by *how*^*24B*^*-GAL4* (Fig 2H). Of note, overexpression of a constitutively active form of AMPKα (AMPKα^T184D^) in the mesoderm resulted in the rescue of *Pngl*^*–/–*^ gut clearance and lethality phenotypes to an extent similar to that of *AMPKα Dp* and *AMPKα*^*WT*^ overexpression (S5A and S5B Fig). Together, these data indicate that a reduction in *AMPKα* expression level in mesodermal cells results in the gut clearance phenotypes observed in *Pngl*^*–/–*^animals and contributes to their lethality. Furthermore, the similarity between the level of rescue conferred to *Pngl*^*–/–*^animals by wild-type and constitutively active forms of AMPKα and the normal pAMPKα/AMPKα ratio in *Pngl*^*–/–*^ midguts strongly suggest that phosphorylation of AMPKα are not impaired in these animals.

### *Pngl*^*–/–*^ midguts show abnormal mitochondrial morphology and impaired energy metabolism, both of which are rescued by restoring *AMPKα* expression

**Fig 3:**
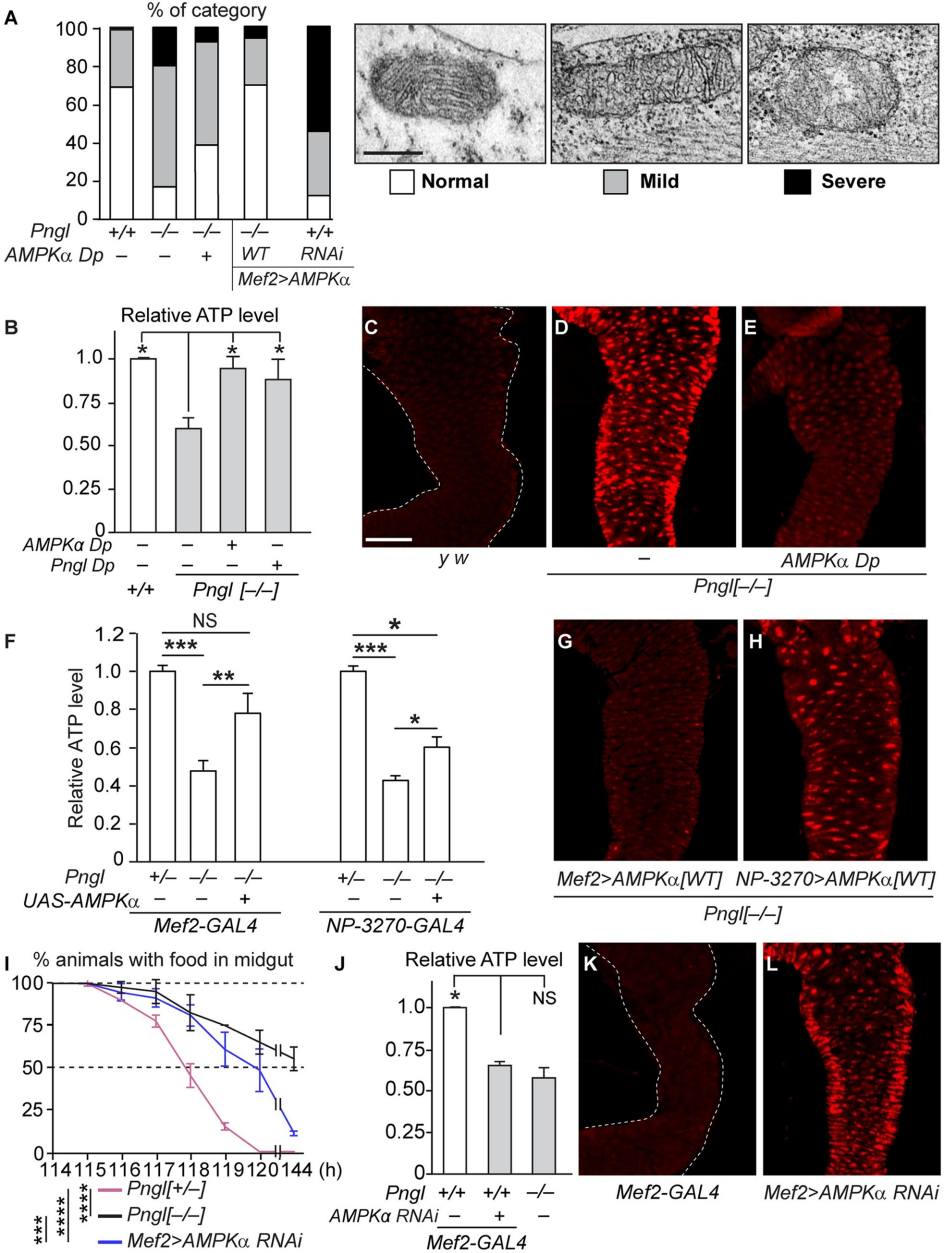
*Pngl* mutant midguts show altered mitochondrial morphology and impaired energy metabolism caused by reduced *AMPKα* levels. (A) Stacked column graph showing the quantification of different categories of mitochondrial morphology in larval visceral muscles of the indicated genotypes. Transmission electron microscopic images representative of each mitochondrial morphology category are shown to the right. *Pngl*^*–/–*^ visceral muscle shows a high degree of mitochondrial abnormality, which is improved by adding one copy of *AMPKα* and rescued upon *AMPKα* overexpression in the mesoderm. RNAi-mediated knock-down of *AMPKα* in the mesoderm also results in abnormalities in mitochondrial morphology. (B) ATP levels in *Pngl*^*–/–*^ larval midguts are significantly reduced compared to control but are restored by adding one copy of *AMPKα* or *Pngl*. (C-E) Confocal images of larval midguts showing ROS levels probed with dihydroethidium (DHE) in the indicated genotypes. *AMPKα* duplication dramatically reduces ROS levels in *Pngl*^*–/–*^ midguts. (F) Graph showing relative ATP levels in *Pngl*^*–/–*^ larvae with overexpression of *AMPKα* using *Mef2-GAL4* and *NP-3270-GAL4*. (G-H) Confocal images showing ROS levels in *Pngl*^*–/–*^ larvae with overexpression of *AMPKα* using *Mef2-GAL4* and *NP-3270-GAL4*. Note that the rescue of ATP levels and ROS accumulation is more robust upon mesodermal expression compared to endodermal expression. (I-L) *AMPKα* RNAi using *Mef2-GAL4* results in food accumulation (I), reduced ATP levels (J), and increased ROS (K) in a wild-type background, indicating that reduced *AMPKα* expression level can explain these phenotypes in *Pngl* mutants. The x-axis in I shows hours (h) after egg laying. Data in B, F, I, and L represent mean ± SD of three independent experiments. Significance is ascribed as *P<0.05, **P<0.01, ***P<0.001, and ****P<0.0001. NS, not significant. Scale bar in (A) is 300 nm and applies to all three TEM images in this panel. Scale bar in (C) is 50 μm and applies to all confocal images in this figure.

Previous work in *C*. *elegans* and mammalian cells has suggested that loss of NGLY1 leads to a reduction in mitochondrial oxidative phosphorylation and an increase in oxidative stress [12]. Therefore, we examined whether *Pngl*^*–/–*^ larvae exhibit any defects in mitochondrial morphology and energy metabolism and if yes, whether these defects can be improved by increasing *AMPKα* levels. We performed TEM on control and mutant midguts and classified the visceral muscle mitochondrial morphology into three different categories: normal, mildly damaged (disorganized cristae structures), and severely damaged (disrupted cristae structures) (Fig 3A, TEM images). Based on this analysis, around 70% of mitochondria in wild-type larvae looked normal, with the remaining 30% mostly showing mild damage (Fig 3A). However, less than 20% of mitochondria in *Pngl*^*–/–*^ larval midgut musculature were categorized as normal, with 60% mildly damaged and ~20% severely damaged (Fig 3A). The overall mitochondrial morphology in *Pngl*^*–/–*^ larvae is improved by adding a genomic copy of *AMPKα* and more so upon mesodermal overexpression of *AMPKα*^*WT*^, strongly suggesting that reduced AMPKα level might contribute to this phenotype.

In agreement with mitochondrial defects reported for *Ngly1* mutants in other organisms [12,13], *Pngl*^*–/–*^ midguts exhibited a statistically significant reduction in ATP levels (Fig 3B). One copy of the *AMPKα Dp* fully rescued this phenotype, similar to one copy of *Pngl Dp* (Fig 3B). Loss of *Pngl* also induced a high level of oxidative stress in the larval midgut, as evidenced by a dramatic increase in the level of oxidized-dihydroethidium (DHE) fluorescence, which is an indicator of reactive oxygen species (ROS) (Fig 3C and 3D). Again, adding one genomic copy of *AMPKα* strongly reduced the ROS marker in *Pngl*^*–/–*^ midguts (Fig 3E). The *AMPKα Dp* is likely to increase *AMPKα* expression in all cell types that normally express this gene. Therefore, the rescue conferred by this reagent did not allow us to distinguish the contribution of *AMPKα* expressed in midgut visceral musculature versus endodermal cells to the observed phenotypes. To address this issue, we performed cell type-specific rescue experiments. Overexpression of *AMPKα*^*WT*^ in *Pngl*^*–/–*^ larvae using a mesodermal driver fully rescued ATP levels and resulted in a strong reduction in ROS levels (Fig 3F and 3G). However, only a modest rescue in ATP level and a mild reduction in ROS levels were observed upon endodermal overexpression of *AMPKα*^*WT*^ (Fig 3F and 3H). These observations suggest that reduced *AMPKα* expression in the mesoderm contributes to the impaired energy homeostasis observed in *Pngl*^*–/–*^ larval midguts.

Using a null allele of *AMPKα* and transgenic overexpression studies, it was previously reported that expression of *AMPKα* in the visceral mesoderm is required for midgut peristalsis [20]. If a reduction in the mesodermal expression of *AMPKα* plays a causative role in the impaired energy homeostasis in *Pngl*^*–/–*^ midguts, we would expect to see similar phenotypes upon *AMPKα* knock-down in a *Pngl*^*+/+*^ background. To examine this notion, we used a previously verified *UAS-AMPKα*^*RNAi*^ strain [21] to decrease its expression in the mesoderm. In agreement with the above-mentioned report [20], *AMPKα* RNAi in the mesoderm resulted in abnormal mitochondrial morphology (Fig 3A) and impaired gut clearance (Fig 3I). Moreover, midguts from animals with mesodermal *AMPKα* RNAi showed a reduction in ATP level and an increase in ROS comparable to that observed in *Pngl*^*–/–*^ midguts (Fig 3J–3L). Altogether, these data support the conclusion that reduced *AMPKα* expression in the visceral mesoderm plays a major role in the impaired energy homeostasis and gut clearance phenotype observed in *Pngl*^*–/–*^ animals, which contribute to the lethality observed in these animals.

### Reduced *AMPKα* expression and impaired energy homeostasis in *Ngly1*^*–/–*^ mouse embryonic fibroblasts

**Fig 4:**
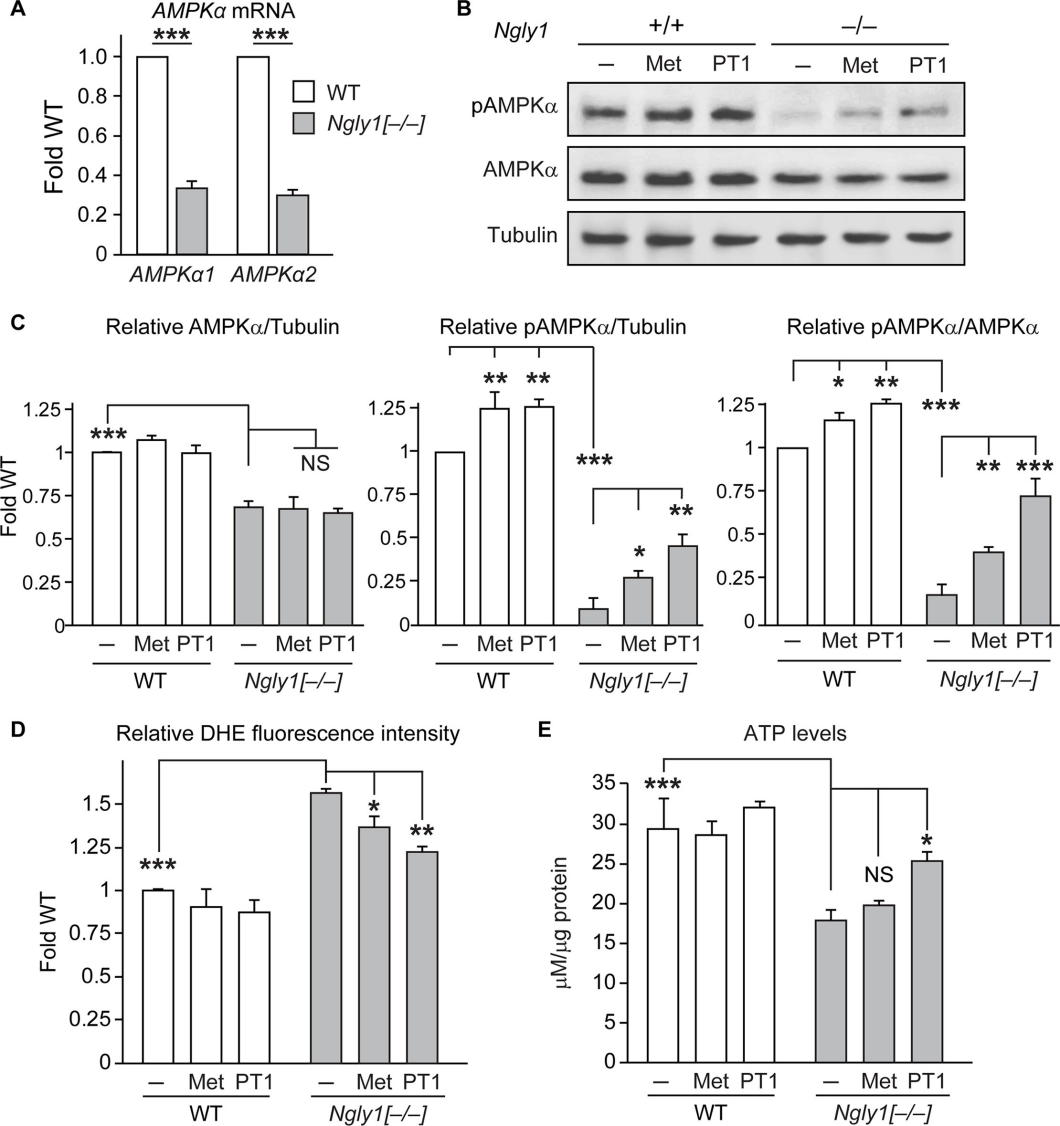
Reduced *AMPKα* level in *Ngly1*^*–/–*^ MEFs and partial rescue of energy metabolism defects in these cells by AMPK activators. (A) qRT-PCR assays show reduced *AMPKα1 and AMPKα2* mRNA expression levels in *Ngly1*^*–/–*^ MEFs compared to wild-type MEFs. (B-D) Western blots show reduced levels of AMPKα and pAMPKα proteins in *Ngly1*^–/–^ MEFs. Treatment of AMPK activators metformin (Met) and PT1 increased pAMPKα levels without affecting AMPKα levels. Graphs show relative levels of (C) AMPKα/Tubulin, pAMPKα/Tubulin and pAMPKα/AMPKα ratios based on quantification of Western blots. (D-E) Graphs show relative ROS levels in terms of oxidized-DHE fluorescent intensity (D) and ATP levels (E) in the indicated cells and conditions. Data represent mean ± SD of three independent experiments. Significance is ascribed as *P<0.05, **P<0.01 and ***P<0.001. NS, not significant.

To examine whether AMPKα plays a conserved role downstream of NGLY1, we used *Ngly1*^*–/–*^ mouse embryonic fibroblasts (MEFs) established from animals homozygous for the *Ngly1*^*tm1*.*1Tasuz*^ allele [22]. Mammalian genomes harbor two *AMPKα* genes: *AMPKα1* (*Prkaa1*) and *AMPKα2* (*Prkaa2*) [23]. qRT-PCR experiments showed a significant reduction in the expression of both genes in *Ngly1*^*–/–*^ MEFs compared to controls (Fig 4A) but not in the mRNA levels of *AMPKβ1*, *AMPKβ2*, *AMPKγ1* and *AMPKγ2* (S6 Fig). Immunoblotting with antibodies that detect both α1 and α2 proteins [24] showed a significant reduction in AMPKα levels and the pAMPKα/AMPKα ratio in *Ngly1*^*–/–*^ MEFs (Fig 4B and 4C). These observations suggest that in addition to reduced AMPKα expression, AMPKα phosphorylation might also be impaired in *Ngly1*^*–/–*^ MEFs. Indeed, the mRNA level of liver kinase B1 (*Lkb1*), which encodes a major AMPKα kinase [17], shows a modest but statistically significant reduction in *Ngly1*^*–/–*^ MEFs (S6 Fig), suggesting a potential mechanism for reduced AMPKα phosphorylation in these cells.

Similar to midguts from *Pngl*^*–/–*^ larvae, *Ngly1*^*–/–*^ MEFs showed a significant increase in the level of ROS and a significant reduction in the level of ATP (Fig 4D and 4E). We next examined whether increasing AMPK signaling can improve these phenotypes in *Ngly1*^*–/–*^ MEFs. For these experiments, we used a pharmacological approach and incubated the cells with two different compounds known to activate AMPK signaling: metformin and PT1 [25–27]. Incubation of both wild-type and *Ngly1*^*–/–*^ MEFs with each of these compounds resulted in a significant increase in the pAMPKα levels without changing the AMPKα expression level (Fig 4B and 4C). PT1 was more effective than metformin in increasing pAMPKα levels in *Ngly1*^*–/–*^ MEFs, although it showed a comparable effect in control MEFs (Fig 4B and 4C). Both compounds reduced oxidative stress in *Ngly1*^*–/–*^ MEFs (Fig 4D). Moreover, PT1, but not metformin, increased the ATP levels in *Ngly1*^*–/–*^ MEFs (Fig 4E), potentially because PT1 directly activates AMPK signaling but metformin indirectly activates AMPK signaling by interfering with the function of mitochondrial respiratory chain complex I [25,26]. Together, these results suggest that reduced AMPK signaling contributes to impaired energy homeostasis in *Ngly1*^*–/–*^ MEFs.

### The impaired energy metabolism and reduced AMPKα levels in *Ngly1/Pngl* mutant contexts are not due to NFE2L1-related proteasome and mitophagy defects

Previous work in *C*. *elegans* and mammalian cell lines has shown that NGLY1 (PNG-1 in worms) is essential for deglycosylation and activation of NFE2L1/SKN-1 [9–11]. In response to proteasome dysfunction, NFE2L1/SKN-1 is activated and induces the transcription of proteasomal genes to restore proteasome function, a process called the “proteasome bounce-back response” [10,11,28]. Transcriptome analysis of adult *Drosophila* upon RNAi-mediated *Pngl* knock-down also showed proteasomal gene downregulation [29]. In addition, *Pngl*^–/–^ larvae were 25 times more sensitive to the toxic effects of the proteasome inhibitor bortezomib compared to *Pngl*^*+/–*^ larvae [30]. These observations indicated that similar to its mammalian and worm homologs, *Pngl* promotes proteasomal gene expression in flies. Given the decreased *AMPKα* expression and pAMPKα levels in both *Pngl*^*–/–*^ larval midguts and *Ngly1*^*–/–*^ MEFs observed in our experiments, we asked whether impaired NFE2L1 activation and proteasome activity contribute to these phenotypes.

**Fig 5:**
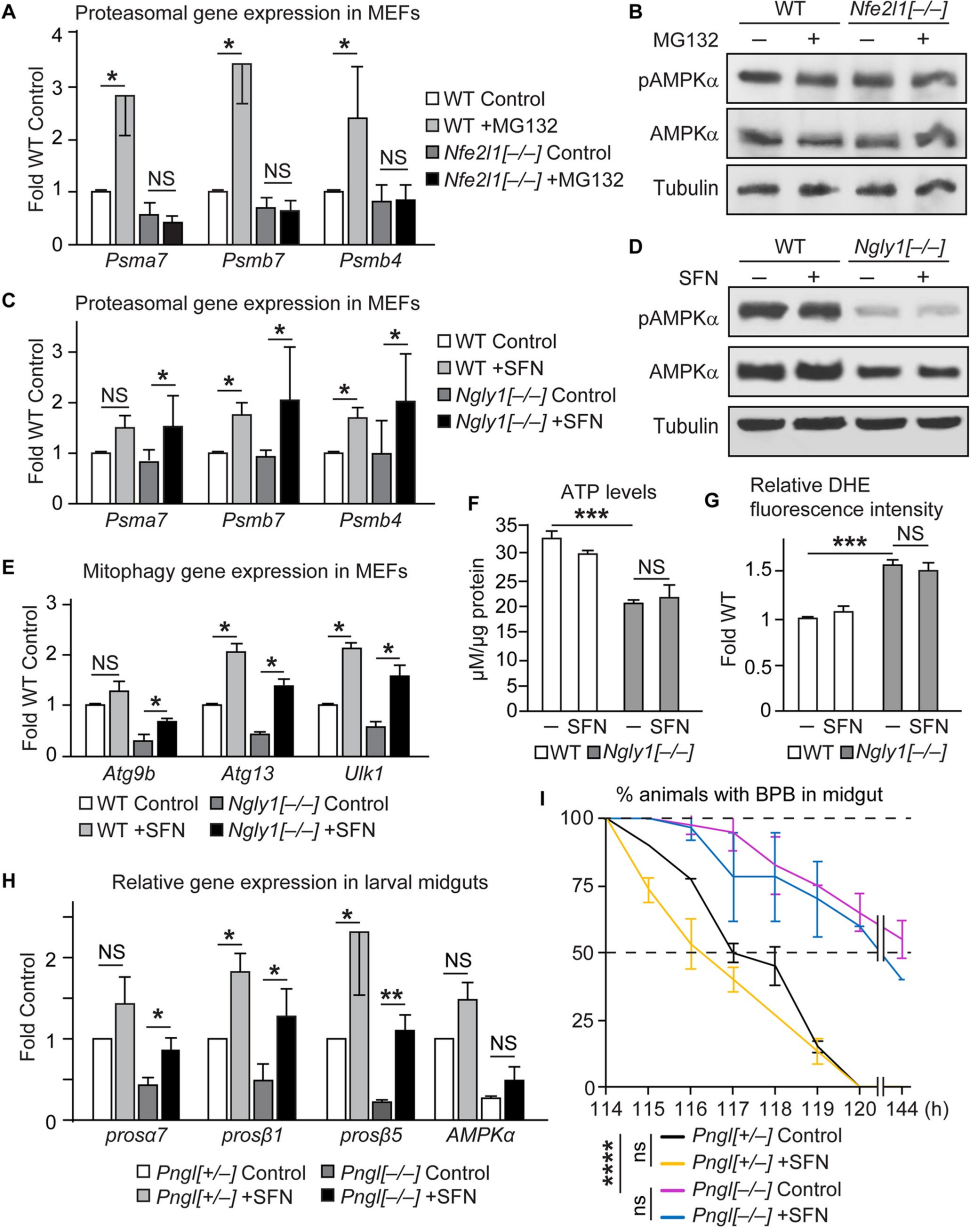
The AMPKα defects in *Ngly1*/*Pngl* mutants are not caused by impaired NGLY1- NFE2L1-proteasomal bounce-back pathway. (A) qRT-PCR assays show relative mRNA levels of proteasomal (Psm) genes in control and MG132-treated wild-type and *Nfe2l1*^*–/–*^ MEFs. Note the impaired proteasome bounce-back response in *Nfe2l1*^*–/–*^ MEFs. (B) Western blots show that loss *of Nfe2l1* and/or treatment with MG132 do not reduce the level of AMPKα and pAMPKα in MEF cells. (C) qRT-PCR assays show relative mRNA levels of Psm genes in control and Sulforaphane (SFN)-treated wild type and *Ngly1*^*–/–*^ MEFs. Note the rescue of the proteasome bounce-back response in *Ngly1*^*–/–*^ MEFs by SFN. (D) Western blots show that SFN treatment does not increase the level of AMPKα and pAMPKα in *Ngly1*^*–/–*^ MEFs. (E) SFN treatment significantly increases the expression of mitophagy genes in *Ngly1*^*–/–*^ MEFs. (F-G) SFN treatment does not rescue energy metabolism defects in *Ngly1*^*–/–*^ MEFs. Graphs show relative levels of ATP (F) and ROS (G) in the indicated cell lines with or without SFN treatment. (H) qRT-PCR assays show relative mRNA levels of Psm genes and *AMPKα* in midguts of control and SFN-treated *Pngl*^*+/–*^ and *Pngl*^*–/–*^ larvae. Note that SFN restores normal Psm gene expression but not *AMPKα* expression in *Pngl*^*–/–*^ larval midguts. (I) Gut clearance assays show that SFN treatment cannot rescue the food accumulation phenotype in *Pngl*^*–/–*^ larvae. The x-axis shows hours (h) after egg laying. All data represent mean ± SD of three independent experiments. Significance is ascribed as *P<0.05, **P<0.01, ***P<0.001, and ****P<0.0001. NS, not significant.

In agreement with a previous report [28], loss of *Nfe2l1* in MEFs impaired the proteasome bounce-back response normally seen upon treating the cells with the proteasome inhibitor MG132 (Fig 5A). This is very similar to the impaired proteasome bounce-back response observed in *Ngly1*^*–/–*^ MEFs upon bortezomib treatment (S7A Fig) and is in agreement with the notion that impaired NFE2L1 activation underlies the proteasome dysfunction observed upon loss of *Ngly1* and its worm homolog [9,11]. However, *Nfe2l1*^*–/–*^ MEFs did not show any decrease in AMPKα and pAMPKα levels (Fig 5B). Moreover, MG132 treatment did not alter the AMPKα and pAMPKα levels in control and *Nfe2l1*^*–/–*^ MEFs (Fig 5B). It has recently been shown that a chemical activator of the NFE2L1 homolog NFE2L2 called sulforaphane (SFN) [31] is able to bypass the loss of NFE2L1 activity in *Ngly1*^*–/–*^ MEFs and restore the proteasome bounce-back response in these cells [13]. As expected, SFN treatment led to a robust increase in proteasomal gene expression in *Ngly1*^*–/–*^ and control cells (Fig 5C). However, this was not accompanied by an increase in the mRNA (S7B Fig) and protein levels of AMPKα and pAMPKα in SFN-treated *Ngly1*^*–/–*^ cells (Fig 5D). SFN treatment has also been shown to promote mitophagy in *Ngly1*^*–/–*^ MEFs by enhancing the expression of mitophagy-related genes [13]. In agreement with this report, we observed a robust increase in the expression of mitophagy-related genes in *Ngly1*^*–/–*^ MEFs treated with SFN (Fig 5E). However, neither ATP nor ROS levels were rescued in these cells upon SFN treatment (Fig 5F and 5G). Together, these data suggest that the reduced AMPKα expression and impaired energy metabolism in *Ngly1*^*–/–*^ MEFs are not due to NFE2L1-related defects in the expression of proteasome and mitophagy genes.

We next performed similar experiments in *Drosophila*. Compared to control larvae, *Pngl*^*–/–*^ larvae showed a strong reduction in the expression of a number of proteasomal genes even without treatment with a proteasomal inhibitor (Fig 5H). These data are in agreement with RNA-seq results from adult *Pngl* knock-down animals [29] and indicate a critical role for Pngl in maintaining proteasomal gene expression in flies. Adding SFN to the fly food resulted in a robust increase in proteasomal gene expression in both control and *Pngl*^*–/–*^ larval midguts and restored the expression of these genes in *Pngl*^*–/–*^ midguts to levels comparable to those in control animals without SFN (Fig 5H). However, SFN was not able to restore *AMPKα* gene expression in *Pngl*^*–/–*^ midguts (Fig 5H). Moreover, the SFN-fed *Pngl*^*–/–*^ larvae still showed impaired gut clearance (Fig 5I). Finally, SFN did not rescue the lethality of *Pngl*^*–/–*^ animals beyond the ~1% Mendelian ratio usually observed in these animals (n = 150). Altogether, the MEF and *Drosophila* data provide compelling evidence that the reduced *AMPKα* expression observed upon loss of *Ngly1/Pngl* and its functional consequences are not due to impaired NFE2L1 activation.

### Reduced *AMPKα* expression and reduced cellular respiration in *NGLY1* deficiency patient fibroblasts

**Fig 6:**
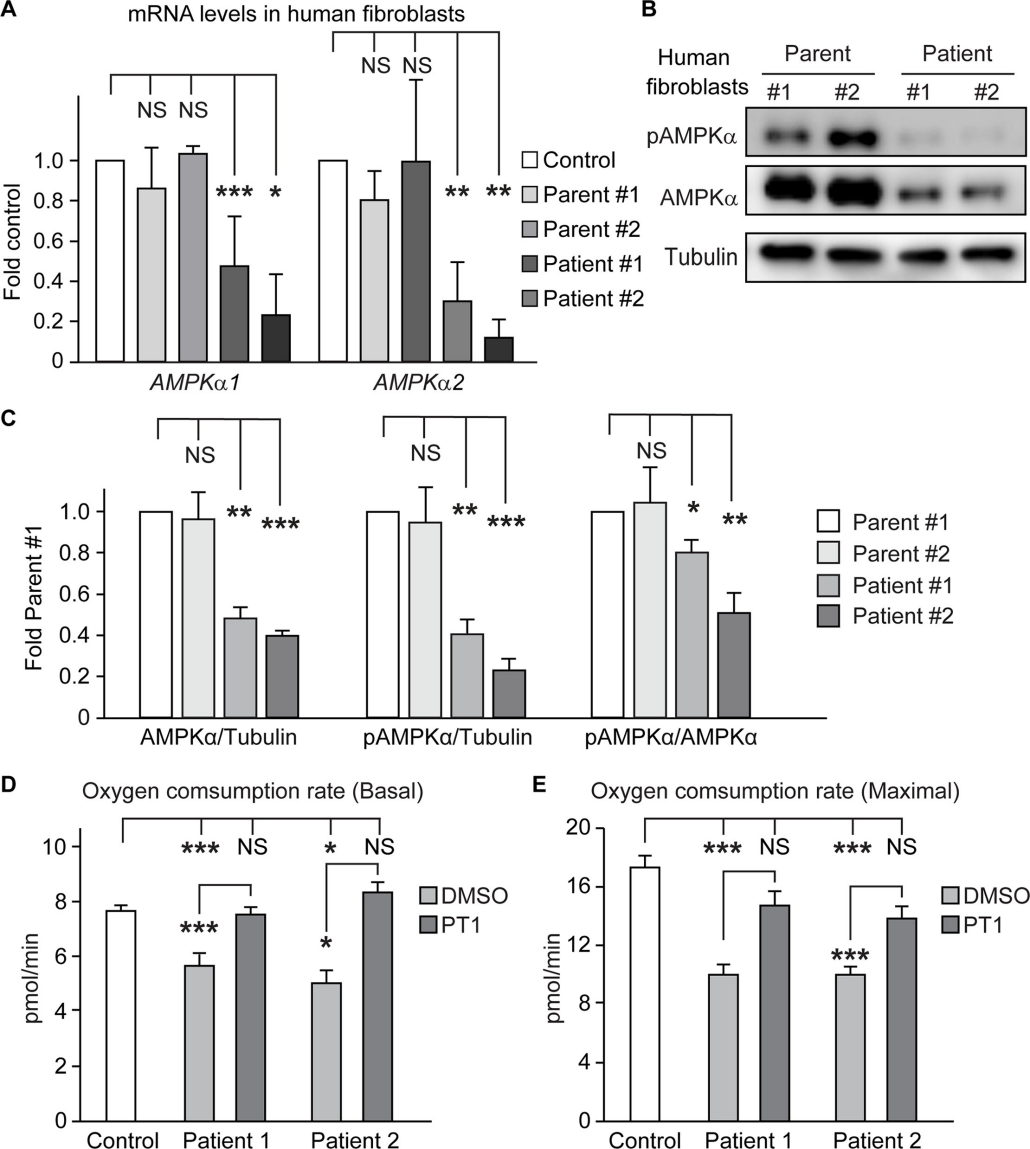
Decreased AMPKα expression in *NGLY1* deficiency patient fibroblasts and rescue of impaired cellular respiration in these cells by PT1 treatment. (A) qRT-PCR assays show a significant decrease in the mRNA levels of *AMPKα1* and *AMPKα2* in two different *NGLY1* deficiency patient fibroblasts compared to control (*NGLY1*^*+/+*^) and two different parents (*NGLY1*^*+/–*^) fibroblasts. (B) Western blots show a severe reduction in AMPKα and pAMPKα protein levels in two different *NGLY1* deficiency patient fibroblasts compared to two independent heterozygous fibroblasts from parents. (C) Graphs showing AMPKα/tubulin, pAMPKα/tubulin and pAMPKα/AMPKα ratios based on quantification of Western blots in (B) and two other independent experiments. (D-E) Basal (D) and maximal (E) oxygen consumption rates are reduced in *NGLY1* deficiency patient fibroblasts compared to control (*NGLY1*^*+/+*^) fibroblasts and are rescued upon PT1 treatment. Data represent mean ± SD of three independent experiments. Significance is ascribed as *P<0.05, **P<0.01 and ***P<0.001. NS, not significant.

To examine whether *AMPKα* expression and AMPK activation (measured by pAMPKα level) are also affected in *NGLY1* deficiency patients, we performed qRT-PCR and immunoblotting on two patients with different *NGLY1* allelic combinations and control fibroblasts. Similar to the other models examined in this study, a significant decrease was observed in the expression levels of *AMPKα1* and *AMPKα2*, but not *AMPKβ1*, *AMPKβ2*, *AMPKγ1* and *AMPKγ2*, in *NGLY1* deficiency patient fibroblasts compared to *NGLY1*^*+/+*^ fibroblasts isolated from a healthy control and *NGLY1*^*+/–*^ fibroblasts isolated from two *NGLY1* deficiency parents (Fig 6A and S8A Fig). Furthermore, both AMPKα and pAMPKα levels and the ratio of pAMPKα/AMPKα in *NGLY1* deficiency patient fibroblasts are significantly decreased compared to parent fibroblasts (Fig 6B and 6C). These data indicate that regulation of *AMPKα* expression is an evolutionarily conserved function of NGLY1. The data also suggest that similar to *Ngly1*^*–/–*^ MEFs, patient fibroblasts might be defective in AMPKα phosphorylation. Of note, unlike *Ngly1*^*–/–*^ MEFs, the patient fibroblasts do not show reduced *LKB1* levels (S8B Fig).

To examine whether *NGLY1* deficiency patient fibroblasts exhibit defects in mitochondrial respiration, we evaluated the oxygen consumption rate (OCR) in patient and control fibroblasts. The basal OCR was significantly reduced in both patient fibroblasts compared to an *NGLY1*^*+/+*^ control (Fig 6D). Treating the patient cells with PT1 fully rescued the basal OCR in both cell lines. The patient cells also showed a significant reduction in maximal OCR, which was rescued by PT1 treatment in both cell lines (Fig 6E). Together, these observations suggest that reduced *AMPKα* expression contributes to the cellular respiration defects in *NGLY1* deficiency patients and could be considered as a target to improve this phenotype.

## Discussion

The discovery of recessive *NGLY1* mutations in patients with a multi-system developmental disorder has prompted a series of studies on the consequences of loss of this enzyme in several cellular and animal model systems. Perhaps the most-studied cellular process downstream of NGLY1 is the NFE2L1-mediated regulation of the proteasomal gene expression, which depends on NGLY1 in both invertebrates and mammals [9–11,29,30]. In addition, our work in *Drosophila*, MEFs and mouse embryos has identified the *Drosophila* Dpp and its mammalian homolog BMP4 as direct, biologically relevant targets of Pngl/NGLY1 and has shown that impaired BMP signaling in the visceral mesoderm of *Pngl*^*–/–*^ larvae results in specific developmental abnormalities in their midgut and contributes to their lethality [16,19]. Our current data indicate that loss of *N*-glycanase 1 also results in a significant reduction in the level of *AMPKα* mRNA in the fly larval midgut, MEFs, and human patient fibroblasts, accompanied by reduced AMPKα and pAMPKα protein levels in all three models. We had previously reported that in addition to the tissue-specific loss of BMP signaling in the midgut, *Pngl*^*–/–*^ larvae exhibit a food accumulation phenotype (failure to empty the gut) that cannot be explained by loss of BMP signaling [16]. We now show that this phenotype is associated with impaired energy homeostasis in the midgut, and that restoring *AMPKα* expression rescues the food accumulation phenotype and allows 40–45% of *Pngl*^*–/–*^ larvae to reach adulthood, compared to ~1% without rescue. In addition, *AMPKα* RNAi in the mesoderm recapitulates the gut clearance and energy homeostasis phenotypes of *Pngl*^*–/–*^ larvae. Moreover, pharmacological activation of AMPK signaling significantly improves the energy homeostasis defects observed in NGLY1-deficient MEFs and patient fibroblasts. Together, these observations suggest that reduced AMPK signaling underlies some of the phenotypes observed in NGLY1-deficient models. It is worth noting that in *Ngly1*^*–/–*^ MEFs and patient fibroblasts, but not in *Drosophila* visceral mesoderm, the pAMPKα/AMPKα ratio was also decreased compared to controls. These observations suggest that in addition to reduced *AMPKα* levels, NGLY1-deficient mammalian cells might also be defective in AMPKα phosphorylation (activation).

Loss of *N*-glycanase 1 in worms, MEFs, and fibroblasts and muscle biopsies from *NGLY1* deficiency patients results in functional abnormalities in mitochondria, including a reduction in oxidative phosphorylation, basal OCR and maximal OCR, and an increase in oxidative stress [7,12,13]. Moreover, *Ngly1*^*–/–*^ MEFs showed mitochondrial fragmentation [13]. In agreement with these observations, we found that mitochondria in the midgut visceral muscles of *Pngl*^*–/–*^ larvae exhibit abnormal cristae structure, accompanied by reduced ATP levels and increased oxidative stress. Importantly, increasing the *AMPKα* expression level in *Pngl*^*–/–*^ animals partially rescued the mitochondrial morphology in the visceral muscle, significantly reduced the level of reactive oxygen species, and fully restored the ATP levels in the midgut. Moreover, pharmacological activation of AMPKα reduced reactive oxygen species and increased ATP levels in MEFs, and restored the basal and maximal OCR in fibroblasts from *NGLY1* deficiency patients. Together, these observations indicate that enhancing AMPK signaling improves mitochondrial energy metabolism in several *N*-glycanase 1-deficient contexts.

Given the impairment of NFE2L1-mediated gene regulation in all *N*-glycanase 1-deficient animal models tested so far, we hypothesized that the reduction in *AMPKα* levels observed in our models might result from the loss of NFE2L1’s transcriptional activity. However, *Nfe2l1*^*–/–*^ and wild-type MEFs showed comparable levels of AMPKα and pAMPKα, despite the impaired proteasome bounce-back response in *Nfe2l1*^*–/–*^ MEFs. Moreover, although treatment with the NFE2L2 activator SFN increased the expression of proteasomal genes in the midguts of *Pngl*^*–/–*^ larvae and in *Ngly1*^*–/–*^ MEFs, it failed to restore normal AMPKα levels in these models. Lastly, treating wild-type MEFs with a proteasome inhibitor did not affect AMPKα and pAMPKα levels. Together, these data indicate that the reduced *AMPKα* observed in NGLY1-deficient contexts is not caused by loss of NFE2L1 activity or impaired proteasome function. The mitochondrial fragmentation phenotype observed in *Ngly1*^*–/–*^ MEFs was shown to primarily result from impaired mitophagy due to loss of NFE2L1 activity and was significantly improved upon treating the cells with SFN [13]. However, SFN treatment failed to suppress the energy metabolism defects in *Ngly1*^*–/–*^ MEFs, even though it rescued the mitophagy gene expression in these cells. Finally, adding SFN to the fly food did not lead to any rescue of the AMPKα-dependent food accumulation and lethality in *Pngl*^*–/–*^ larvae. Taken together, our data suggest that the impairment of energy metabolism observed upon loss of NGLY1 is caused by reduced AMPKα activity and is independent of NFE2L1-related defects in proteasome and mitophagy.

Our previous report [16] and current data (S3 Fig) demonstrate that the loss of BMP signaling observed in *Pngl*^*–/–*^ midguts does not cause the food accumulation phenotype in these animals. Moreover, the data presented in the current study indicate that loss of NFE2L1 activation cannot explain the reduced *AMPKα* expression in *NGLY1*-deficient models. These data suggest that NGLY1 regulates AMPKα levels independently of BMP and NFE2L1 pathways. The mechanisms that regulate the level of *AMPKα* mRNA are not well understood. MicroRNA 148b (miR-148b) and miR-301a are reported to negatively regulate AMPKα levels in pancreatic cancer and osteosarcoma cell lines, respectively [32,33]. A recent study has shown that TP63 is recruited to the *AMPKα1* regulatory region and directly activates *AMPKα1* transcription in human mammary gland cells [34]. However, to our knowledge, neither TP63 nor the above-mentioned microRNAs have been linked to NGLY1 so far. In fact, transcription factors and other proteins involved in mRNA stability are not *N*-glycosylated and therefore cannot be direct targets of NGLY1 (with NFE2L1 as a rare exception). How does then loss of NGLY1 lead to reduced *AMPKα* levels? *Ngly1*^*–/–*^ MEFs have been shown to accumulate cytosolic aggregates of a model ERAD substrate harboring *N*-linked *N*-acetylglucosamine monosaccharides (*N*-GlcNAc) [22], a type of glycan normally not seen in the cytosol. Accumulation of proteins harboring *N*-GlcNAc can potentially interfere with the function of *O*-GlcNAc, which is the major type of glycosylation on nucleocytoplasmic proteins and regulates various cellular processes including transcription and mitochondrial activity [35]. Therefore, one possibility is that NGLY1 regulates *AMPKα* level indirectly through impaired *O*-GlcNAc signaling. Another possibility is that NGLY1 is involved in the quality control of an *N*-glycosylated cell surface receptor that is upstream of *AMPKα* transcription. The molecular mechanism through which NGLY1 regulates AMPKα levels remains to be determined.

## Materials and methods

### *Drosophila* strains and genetics

Animals were grown on standard food containing cornmeal, molasses, and yeast at room temperature. The following strains were used in this study: *y w*, *Mef2-GAL4*, *how*^*24B*^*-GAL4* [18], *elav-GAL4*, *Dp(1;3)DC102*, *PBac{DC102}VK33* (*AMPKα* duplication; [36]), *UAS-AMPKα*^*WT*^ and *UAS-AMPKα*^*T184D*^ [37], and *UAS-AMPKα*^*RNAi*^ (*y1 v1; P{TRiP*.*JF01951}attP2*) (Bloomington *Drosophila* Stock Center); *Pngl*^*ex14*^, *Pngl*^*ex18*^, *UAS-Pngl*^*WT*^ and *UAS-Pngl*^*C303A*^ [15], *NP3270-GAL4* [38] (Kyoto *Drosophila* Stock Center); *UAS-attB-NGLY1*^*WT*^*-VK31* [16], *dpp*^*HA*^ and *dpp*^*HA-3NQ*^ [19] and *PBac{Pngl*^*wt*^*}VK31* (*Pngl* duplication, this study).

### Generation of the *Pngl* genomic transgene (duplication)

To generate a *Pngl* genomic rescue transgene, we purchased the bacterial artificial chromosome CH322-03O13 [39] from BACPAC Resources (https://bacpacresources.org/). The 21,853-bp insert present in this construct only contains the full coding and flanking sequences of two genes: *Pngl* and *Actin 42A*. The construct was integrated into the *VK27* docking site on the third chromosome [40]. The injection to generate the transgene was performed by BestGene, Inc.

### Lethality rescue assays (eclosion tests)

To test the lethality rescue, we scored the number of eclosed progeny and calculated the estimated total number based on Mendelian inheritance for each genotype. The observed/expected ratio is reported as a percentage.

### Gut clearance assay

To examine gut clearance, 3rd instar larvae were transferred to standard food mixed with 1% FD&C No. 1 Blue, which is a synthetic food color. Wandering stage larvae were collected from the side of the vial with a paintbrush, transferred to wet Whatman paper in a petri dish, and were monitored for the presence of the blue color in the gut until puparium formation. About 30 larvae were scored for each genotype.

### Midgut contraction assay

To check the frequency of gut contractions, the intact larval midguts from each genotype were dissected in freshly-made PBS at room temperature without disrupting the attached tissues. After dissection, the number of gut contractions per minute was scored under a light microscope by visual inspection. At least 15 animals per genotype were scored.

### qRT-PCR assays

Total RNA was extracted from 3 larval midguts with Trizol (Invitrogen) and dissolved in 25 μL of RNase-free water. cDNA was then synthesized from 1 μg total RNA using amfiRivert II cDNA Synthesis Master Mix (R5500, GenDEPOT), and qPCR was carried out using amfiSure qGreen Q-PCR Master Mix, Low ROX (Q5601, GenDEPOT). Expression levels were normalized to internal control: actin for fly midguts and MEFs, and GAPDH for human fibroblasts. The following oligonucleotide sequences were used to assess target genes expression (5' to 3'; f, fly; m, mouse; h, human). The following primer pairs were obtained from previous reports: fly *AMPKγ* [41], mouse proteasomal and mitophagy genes [13], mouse and human *AMPK* subunits [42], mouse *Lkb1* [43], and human *LKB1* [44].

f-prosα7-F TTTTCGCCTGATGGCCGCG
f-prosα7-R ACCGGTTACCCTGCCCACCAA
f-prosβ1-F CGAGTCCTGCACCATCGGCG
f-prosβ1-R TGCCAATGCGCACCACACCA
f-prosβ5-F TGGCTGCTCCGCCATTCGAG
f-prosβ5-R CCGGCCAGCATCATGCCCAT
f-AMPKα-F TGGGCACTACCTACTGGG
f-AMPKα-R ATCTGGTGCTCGCCGATCTT
f-AMPKβ-F GCCCTGGGAGAAGGTATCTGG
f-AMPKβ-R AGTGGGTTCACACGATAG
f-AMPKγ-F AAAAACAAAACCAAAAGCAACAA
f-AMPKγ-R AATTATTGGAATTGGAGCTGGAG
f-Actin5C-F TTGTCTGGGCAAGAGGATCAG
f-Actin5C-R ACCACTCGCACTTGCACTTTC
m-Psmb1-F CCTTCAACGGAGGTACTGTATTG
m-Psmb1-R GGGCTATCTCGGGTATGAATTG
m-Psmb4-F CGAGTCAACGACAGCACTAT
m-Psmb4-R ATCTCCCAACAGCTCTTCATC
m-Psma7-F CGAGTCTGAAGCAGCGTTAT
m-Psma7-R AGTCTGATAGAGTCTGGGAGTG
m-AMPK*α*1-F GTCGACGTAGCTCCAAGACC
m-AMPK*α*1-R ATCGTTTTCCAGTCCCTGTG
m-AMPK*α*2-F CGCCTCTAGTCCTCCATCAG
m-AMPK*α*2-R ATGTCACACGCTTTGCTCTG
m-AMPK*β*1-F GTTGCTGTTGCTTGTTCCAA
m-AMPK*β*1-R ATACTGTGCCTGCCTCTGCT
m-AMPK*β*2-F ACCCAAGCACAGCTCTAGACACAA
m-AMPK*β*2-R AGGGTAGTTCCTTGCCTCACACAT
m-AMPK*γ*1-F TCCCTAGACCTCACCACACC
m-AMPK*γ*1-R GTCTGCACAGCACAAGAACC
m-AMPK*γ*2-F ATTGACCCTATCAGTGGGAACGCA
m-AMPK*γ*2-R TCCGATTCCAAGCTCATCCAGGTT
mLkb1-F TTGGGCCTTTTCTCCGAGG
mLkb1-R CAGGTCCCCCATCAGGTACT
m-Atg13-F CTGCTGGGAGGTGCAGTT
m-Atg13-R TTCAGTTTCCATTGCCTGC
m-Atg9b-F CCAGGTGTTTTACAGGGAGG
m-Atg9b-R ACGTCCAGTTCCGTCAGC
m-Ulk1-F ATCGTGGCGCTGTATGACTT
m-Ulk1-R GCAGGTAGTCAGCCAGGTCT
m-Actin-F GGCACCACACCTTCTACAATG
m-Actin-R GGGGTGTTGAAGGTCTCAAAC
h-AMPK*α*1-F CCTCTGCCTAGCACTGCTCT
h-AMPK*α*1-R ATTCCAAAGGTGCCAGTCAG
h-AMPK*α*2-F AGACCAGCTTGCAGTGGCTTATCA
h-AMPK*α*2-R AGAGGTGGCATCCTTTCTGGATGA
h-AMPK*β*1-F TTCCACTCCGAGGAAATCAAGGCA
h-AMPK*β*1-R TGGGCGGGAGCTTTATCATTCACT
h-AMPK*β*2-F TGTGCAGAGAGTGTCAGTTTCCCA
h-AMPK*β*2-R AGGCTTCTCAGAACCATGGGATGT
h-AMPK*γ*1-F CAAGAGACCCCAGAATCCAA
h-AMPK*γ*1-R CCTGCAGGGACGTATCAAAT
h-AMPK*γ*2-F TGCGGCCGAGGATTACATTTATGC
h-AMPK*γ*2-R AAACAAGCCTCCTATGCCTGTGC
h-LKB1-F TCTACACTCAGGACTTCACG
h-LKB1-R GTTCATACACACGGCCTT
h-GAPDH-F GTCTCCTCTGACTTCAACAGCG
h-GAPDH-R ACCACCCTGTTGCTGTAGCCAA

### Cell culture and drug treatment

*Ngly1*^*–/–*^ MEFs were obtained from Dr. Tadashi Suzuki and were authenticated by genotyping [22]. *Nfe2l1*^*–/–*^ MEFs were obtained from Dr. Senthil Radhakrishnan and were verified based on impaired proteasome bounce-back response [28]. Human fibroblasts derived from a healthy 5-year old male control (GM05381) was from Coriell Institute for Medical Research (Camden, NJ) and were kindly provided by Dr. Hud Freeze [45]. The patients and parent fibroblasts had the following genotypes: Parent #1, *NGLY1*^*R401X/+*^; parent #2, *NGLY1*^*R458fs/+*^; patient #1, *NGLY1*^*R401X/R401X*^, patient #2, *NGLY1*^*Q631fs*^*/*^*R401X*^. Cells were cultured in Dulbecco's modified Eagle's medium (Sigma-Aldrich) supplemented with 10% FBS, 1% GlutaMax and antibiotics (100 U/mL penicillin G, 100 ng/mL streptomycin; Sigma-Aldrich) at 37°C in humidified air containing 5% CO_2_ (vol/vol). Bortezomib (BTZ) (Cat. No. 179324-69-7), MG132 (Cat. No. 133407-82-6) and Sulforaphane (SFN) (Cat. No. 4478-93-7) were from Cayman chemicals. Metformin was from Sigma (Cat. No. PHR1084) and PT1 was from TOCRIS (Cat. No. 4039). MEFs were treated with 10 nM BTZ or 1 μM MG132 or 10 μM SFN for 6 hours in a 6-well plate. Cells were incubated in 10 μM Metformin or 10 nM PT1 in the culture media for 3 hours in a 6-well plate.

### Measurement of ATP levels

ATP levels were measured using luciferase-based ATP determination kit (Molecular Probes; A22066) by following the manufacturer’s descriptions. Briefly, larval midgut or MEFs were lysed in homogenizing buffer [6 M guanidine HCL, 100 mM Tris (pH 7.8), 4 mM EDTA] and then boiled for 5 min and centrifuged for 3 minutes at 4°C. Supernatant was diluted (1:500) and 10 μL of it was added to 100 μL of luciferase-reaction buffer to start the reaction in plate reader. Luminescence was measured by a LUMIstar OPTIMA microplate reader (BMG LABTECH). ATP levels were determined by comparing luciferase measurements to ATP standard curve. For relative quantification, ATP levels in different groups were normalized by their respective protein levels.

### Assessment of ROS level

As a measure of oxidative imbalance, ROS levels were detected using the cell permeable dye dihydroethidium (DHE) following an online protocol [46] with minor modifications. When oxidized, DHE forms 2-hydroxyethidium, which intercalates within DNA molecules and generates a bright red fluorescent signal in the nucleus. For the *in vivo* detection of ROS levels by DHE, *Drosophila* larval midguts were dissected out in Schneider’s medium and incubated with 30 μM DHE for 5 minutes in the dark at room temperature. After 1X PBS wash, midguts were mounted and imaged for oxidized DHE under Leica TCS-SP5 microscope. For the detection of ROS levels in cell lines, MEFs were incubated in 15 μM DHE for 15 minutes. After washing in 1X PBS, fluorescence intensity was measured in a FlexStation 3 Microplate reader. Fluorescence intensity was normalized by the number of cells.

### Measurement of the oxygen consumption rate

Oxygen consumption rates (OCR) for cells treated with PT1 and control cells were assessed using the Seahorse XF96 analyzer (Agilent Technologies, CA, USA). Wild-type and patient human fibroblasts were seeded into XF 96-well cell culture plates, and incubated overnight to allow attachment. Maximal OCR was measured after FCCP injection. Cells were treated with the AMPK activator PT1 (10 μM) for 48 hours. Vehicle alone (DMSO) control cells were processed in parallel. After 48 hours of incubation, cells were washed in pre-warmed XF assay media (or for OCR measurement, XF assay media supplemented with 10 mM glucose, 1 mM pyruvate, 2 mM L-glutamine and adjusted at 7.4 pH). Cells were then maintained in 175 μL/well of XF assay media at 37°C, in a CO_2_ incubator for 1 hour. Measurements were normalized by protein content (Bradford assay). The data were analyzed by XF96 software and GraphPad Prism software, using one-way ANOVA and Student's t-test calculations. All experiments were performed in quintuplicate, three times independently.

### Western blotting

Proteins were extracted from larval midguts in lysis buffer containing phosphatase cocktail (Thermo Fisher Cat. No. 78428) and protease inhibitor cocktail (Promega Cat. No. G6521). The following antibodies were used: rabbit anti-pAMPKα 1:1000 (Thr172) (CST Cat. No. 2531), rabbit anti-AMPKα 1:1000 (CST Cat. No. 2532), mouse anti-actin 1:1000 (DSHB Cat. No. 224-236-1), mouse anti-tubulin 1:1000 (Santa Cruz Biotechnology Cat# sc-8035), goat anti-rabbit-HRP and goat anti-mouse-HRP 1:2000 (Jackson ImmunoResearch Laboratories). Western blots were developed using Clarify ECL Western Blotting Substrates (BioRad). The bands were detected using an Azure Biosystems c280 digital imager using chemiluminescent detection of HRP. At least three independent immunoblots were performed for each experiment.

### Assessment of midgut musculature and muscle-mitochondrial morphology

To examine midgut musculature, 3rd instar larval midguts were stained with phalloidin for 1 hour. Confocal images were taken with Leica TCS-SP8 microscope. All images were acquired using Leica LAS-SP software. Amira 5.2.2 and Adobe Photoshop CS6 were used for image processing.

TEM of visceral muscles in larval midguts was performed using a Ted Pella Bio Wave processing microwave with vacuum attachments. Third instar larvae were dissected in 4% paraformaldehyde, 2% glutaraldehyde, 0.1 M sodium cacodylate, and 0.005% CaCl2 (pH 7.2). The samples were incubated in the same fixative for 3 days at 4°C overnight. On the third day, the samples were processed in fix again, post-fixed in 2% OsO4, dehydrated in ethanol series and propylene oxide, and then embedded in Embed-812 resin (Electron Microscopy Sciences, Hatfield, PA). Individual larval midguts were then sectioned and stained in 1% uranyl acetate and 2.5% lead nitrate. TEM images of cross sections were taken using a JEOL JEM 1400 plus transmission electron microscope with an AMT XR-16 mid-mount 16 megapixel digital camera.

Ten TEM Images from 3–5 animals were acquired from randomly selected regions of the 3rd instar larval midguts for each genotype. All mitochondria in these images were scored (*y w*: 5 animals, 117 mitochondria; *Pngl*^*–/–*^: 5 animals, 125 mitochondria; *Pngl*^*–/–*^*; AMPK Dp*: 5 animals, 209 mitochondria; *Mef2>AMPKα RNAi*: 3 animals, 24 mitochondria; *Pngl*^*–/–*^*; Mef2>AMPKα*^*WT*^: 3 animals, 65 mitochondria). The scored mitochondria were grouped into three categories: severe, mild, normal, based on the integrity of the cristae structure, with genotypes blinded to the experimenters, using the ImageJ software (NIH).

### Statistical analysis

Statistical analysis was performed by Student’s t-test or by one-way ANOVA with the Dunnett’s Post hoc multiple comparisons test using GraphPad Prism 6. Curves from gut clearance data were analyzed using Log-rank (Mantel-Cox) test. Data were plotted as mean ± SD of at least three independent experiments. *P values* are mentioned in Figure Legends.

## Supporting information

S1 Fig Schematic of the *Pngl* genomic region and the CH322-03O13 BAC. The full coding and regulatory regions of *Pngl* and *Actin 42A* are present in this BAC, but the 5’UTR and part of the coding region of the other two neighboring genes (*Strica* and *Src42A*) are missing from the BAC.
(TIF)

S2 Fig Loss of *Pngl* does not result in major structural abnormalities in the larval midgut visceral muscle. TEM images of control and *Pngl*^*–/–*^ larval midgut visceral muscle are shown (representative images from n = 4 animals for each genotype, >5 images per animal was examined). Scale bar in (A) is 1 μm and applies to both panels.
(TIF)

S3 Fig The *dpp-* and *AMPKα*-related phenotypes in *Pngl*^*–/–*^ larval midguts are independent from each other. (A) Gut clearance assays in 3rd instar larvae of the indicated genotypes are shown. Note that one copy of the *dpp*^*HA-3NQ*^ does not improve the food accumulation phenotype in *Pngl*^*–/–*^ larvae, even though it fully rescues the *dpp* loss-of-function phenotypes in *Pngl*^*–/–*^ midguts [19]. (B) Bright images of the proximal midgut region of 3rd instar larvae of the indicated genotypes fed with bromophenol blue (BPB) are shown. Asterisks mark gastric caeca; dashed box marks the acid zone, which turns yellow upon BPB feeding. Note that one copy of *AMPKα* duplication (*Dp*) does not rescue the shortened gastric caeca and loss of acid zone in *Pngl*^*–/–*^ midguts, even though it significantly improves the gut clearance and contraction phenotypes (Fig 2). Scale bar in the top panel is 100 μm and applies to all panels.
(TIF)

S4 Fig Restoration of *AMPKα* and AMPKα/pAMPKα levels upon *AMPKα* and *Pngl* duplication and mesodermal overexpression. (A) Graph showing relative expression of *AMPKα* in the larval midguts of the indicated genotypes. Not that mesodermal expression of the enzymatic-deficient *Pngl*^*C303A*^ is not able to rescue *AMPKα* expression in *Pngl*^*–/–*^ midguts. (B) Western blots show that one copy of the *Pngl* duplication and mesodermal overexpression of wild-type *Pngl* but not the catalytically inactive *Pngl*^*C303A*^ restores the level of AMPKα and pAMPKα in *Pngl* mutant midguts. (C) Graph showing relative expression of *AMPKβ and γ* mRNA in larval midguts of the indicated genotypes. NS, not significant. (D) Schematic of the *AMPKα* genomic region and the CH321-61O10 BAC from the P[acman] library [39] used to generate the *AMPKα* duplication *Dp(1;3)DC102*, *PBac{DC102}VK33*. (E) Western blots show that one copy of the *AMPKα* duplication restores the level of AMPKα and pAMPKα in *Pngl* mutant midguts. (F) Graph showing relative levels of *AMPKα* mRNA in larval midguts of the indicated genotypes. Significance is ascribed as *P<0.05 and **P<0.01 compared to control in each panel. NS, not significant.
(TIF)

S5 Fig Rescue of *Pngl*^*–/–*^ food accumulation phenotype and partial rescue of *Pngl*^*–/–*^ lethality by mesodermal overexpression of a constitutively-active form of *AMPKα*. (A) Mesodermal overexpression of *AMPKα*^*T184*^ improves the gut clearance in *Pngl*^*–/–*^ 3rd instar larvae. The x-axis shows hours (h) after egg laying. **P<0.01. (B) Eclosion tests show that mesodermal overexpression of *AMPKα*^*T184*^ rescues the *Pngl*^*–/–*^ lethality by ~42%. Note the rescue achieved by *AMPKα*^*T184*^ is comparable to those achieved by *AMPKα* duplication and *AMPKα*^*WT*^ overexpression (compare to Fig 2). Data in (A) represent mean ± SD of three independent experiments. Animal number from left to right are 200 and 410 in (B).
(TIF)

S6 Fig Loss of *Ngly1* in MEFs does not affect the expression of *AMPKβ1*, *AMPKβ2*, *AMPKγ1* and *AMPKγ2* mRNA but results in a modest reduction in *Lkb1* mRNA level. *P<0.05. NS, not significant.
(TIF)

S7 Fig Impaired proteasome bounce-back response in *Ngly1*^*–/–*^ MEFs. (A) qRT-PCR assays show relative mRNA levels of proteasomal (Psm) genes in control and Bortezomib (BTZ)-treated wild-type and *Ngly1*^*–/–*^ MEFs. Note the impaired proteasome bounce-back response in *Ngly1*^*–/–*^ MEFs, in agreement with a previous report [11]. (B) SFN treatment does not increase the level of *AMPKα1* and *AMPKα2* mRNA in *Ngly1*^*–/–*^ MEFs.
(TIF)

S8 Fig The expression levels of *AMPKβ1*, *AMPKβ2*, *AMPKγ1*, *AMPKγ2* and *LKB1* mRNA are not altered in *NGLY1* deficiency patient fibroblasts compared to control fibroblasts. NS, not significant.
(TIF)
