# Supplementary figures and images for: A conserved role for AMP-activated protein kinase in NGLY1 deficiency
Source: PLoS Genet. 2020 Dec 14;16(12):e1009258. doi: 10.1371/journal.pgen.1009258 (PMC7769621; doi:10.1371/journal.pgen.1009258)

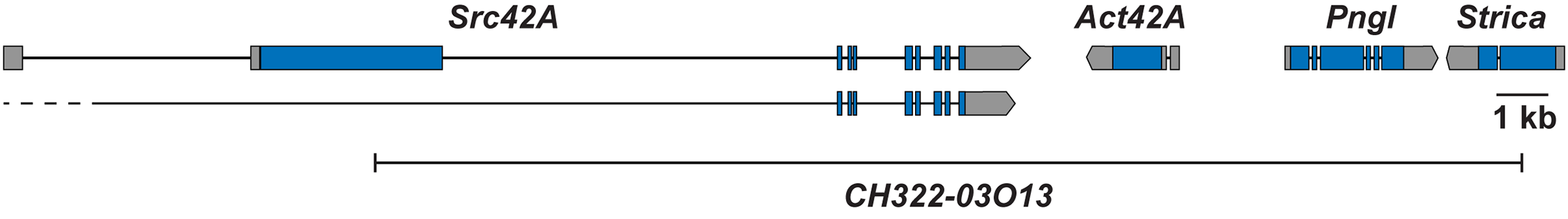

Supplement: S1 Fig — The full coding and regulatory regions of Pngl and Actin 42A are present in this BAC, but the 5’UTR and part of the coding region of the other two neighboring genes (Strica and Src42A) are missing from the BAC. (TIF) [file pgen.1009258.s001.tif]

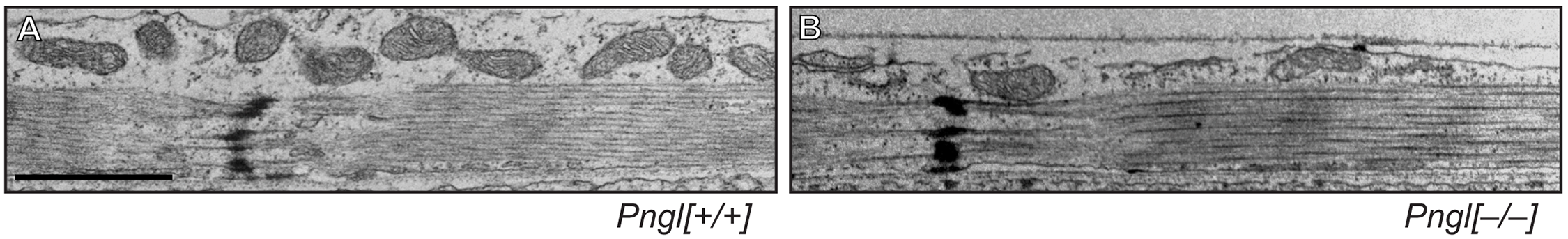

Supplement: S2 Fig — TEM images of control and Pngl–/– larval midgut visceral muscle are shown (representative images from n = 4 animals for each genotype, >5 images per animal was examined). Scale bar in (A) is 1 μm and applies to both panels. (TIF) [file pgen.1009258.s002.tif]

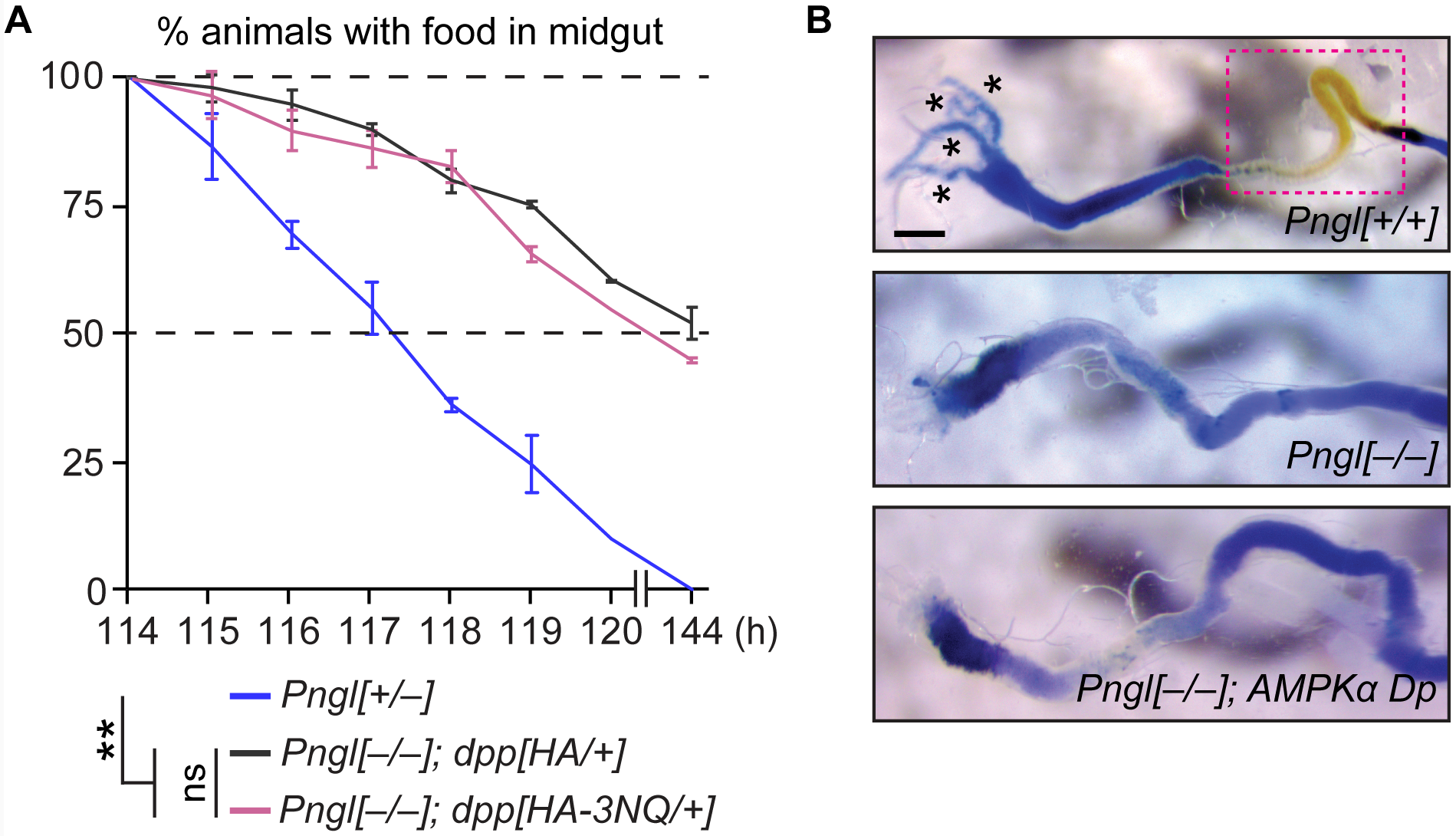

Supplement: S3 Fig — (A) Gut clearance assays in 3rd instar larvae of the indicated genotypes are shown. Note that one copy of the dppHA-3NQ does not improve the food accumulation phenotype in Pngl–/– larvae, even though it fully rescues the dpp loss-of-function phenotypes in Pngl–/– midguts [19]. (B) Bright images of the proximal midgut region of 3rd instar larvae of the indicated genotypes fed with bromophenol blue (BPB) are shown. Asterisks mark gastric caeca; dashed box marks the acid zone, which turns yellow upon BPB feeding. Note that one copy of AMPKα duplication (Dp) does not rescue the shortened gastric caeca and loss of acid zone in Pngl–/– midguts, even though it significantly improves the gut clearance and contraction phenotypes (Fig 2). Scale bar in the top panel is 100 μm and applies to all panels. (TIF) [file pgen.1009258.s003.tif]

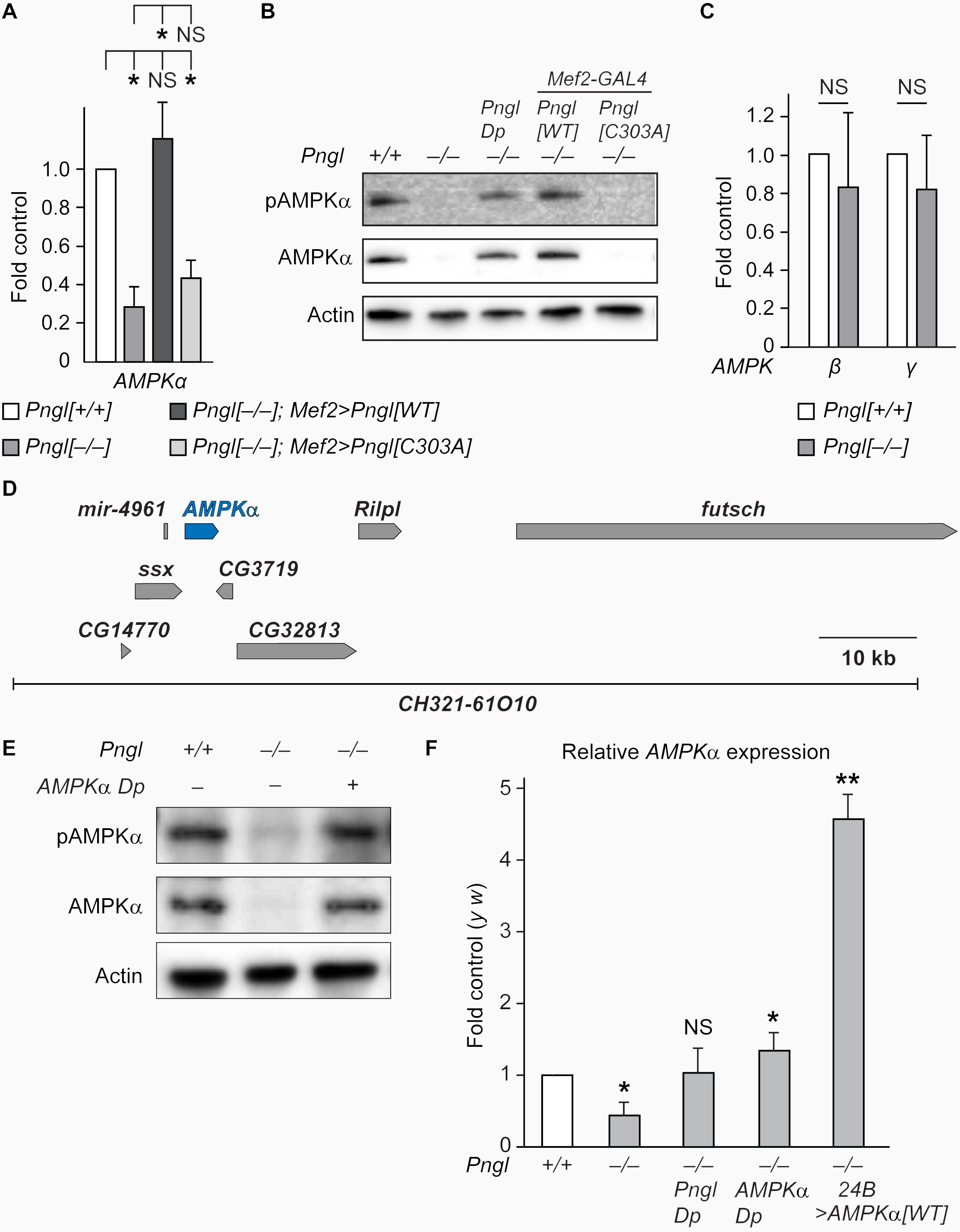

Supplement: S4 Fig — (A) Graph showing relative expression of AMPKα in the larval midguts of the indicated genotypes. Not that mesodermal expression of the enzymatic-deficient PnglC303A is not able to rescue AMPKα expression in Pngl–/– midguts. (B) Western blots show that one copy of the Pngl duplication and mesodermal overexpression of wild-type Pngl but not the catalytically inactive PnglC303A restores the level of AMPKα and pAMPKα in Pngl mutant midguts. (C) Graph showing relative expression of AMPKβ and γ mRNA in larval midguts of the indicated genotypes. NS, not significant. (D) Schematic of the AMPKα genomic region and the CH321-61O10 BAC from the P[acman] library [39] used to generate the AMPKα duplication Dp(1;3)DC102, PBac{DC102}VK33. (E) Western blots show that one copy of the AMPKα duplication restores the level of AMPKα and pAMPKα in Pngl mutant midguts. (F) Graph showing relative levels of AMPKα mRNA in larval midguts of the indicated genotypes. Significance is ascribed as *P<0.05 and **P<0.01 compared to control in each panel. NS, not significant. (TIF) [file pgen.1009258.s004.tif]

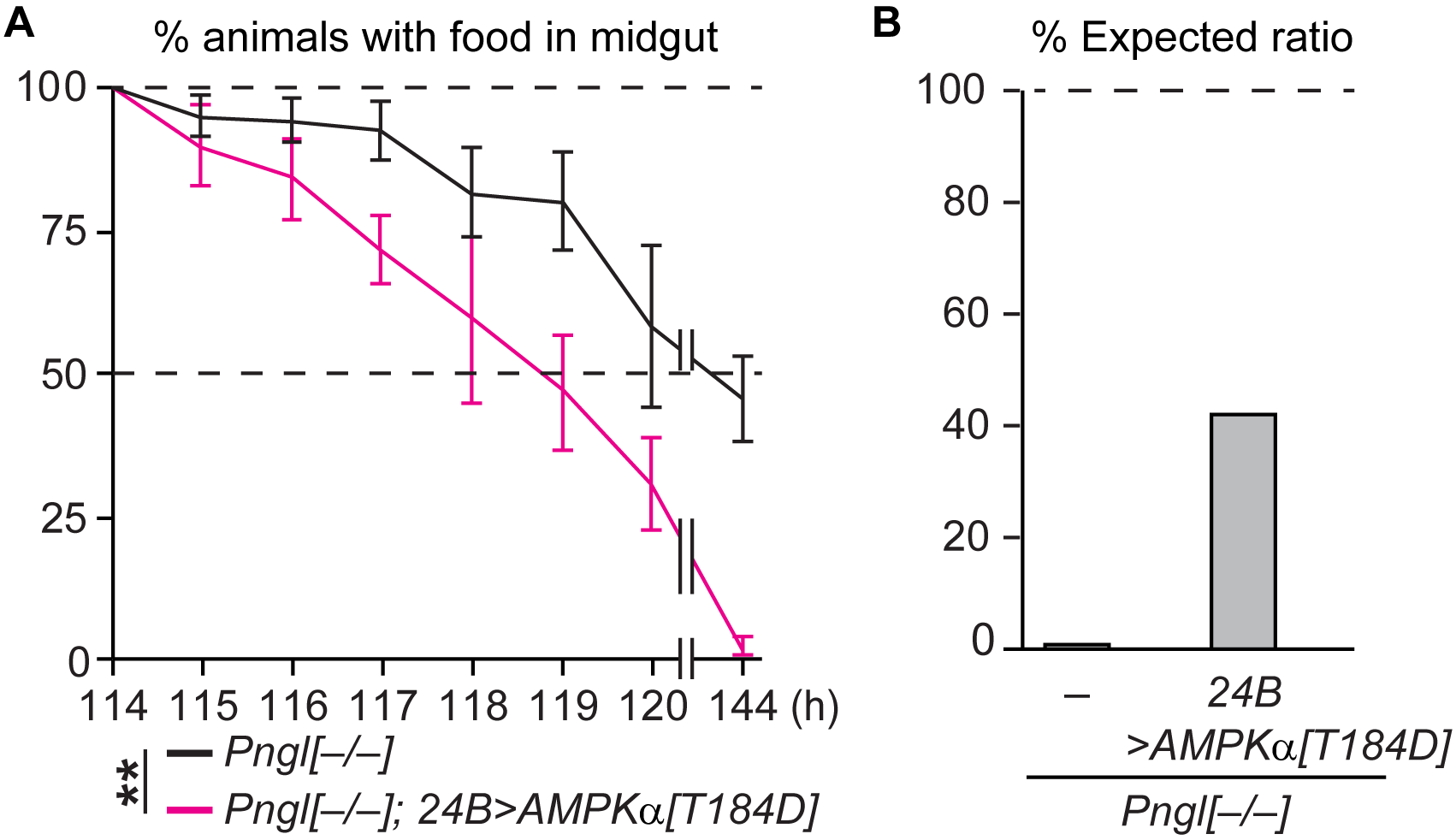

Supplement: S5 Fig — (A) Mesodermal overexpression of AMPKαT184 improves the gut clearance in Pngl–/– 3rd instar larvae. The x-axis shows hours (h) after egg laying. **P<0.01. (B) Eclosion tests show that mesodermal overexpression of AMPKαT184 rescues the Pngl–/– lethality by ~42%. Note the rescue achieved by AMPKαT184 is comparable to those achieved by AMPKα duplication and AMPKαWT overexpression (compare to Fig 2). Data in (A) represent mean ± SD of three independent experiments. Animal number from left to right are 200 and 410 in (B). (TIF) [file pgen.1009258.s005.tif]

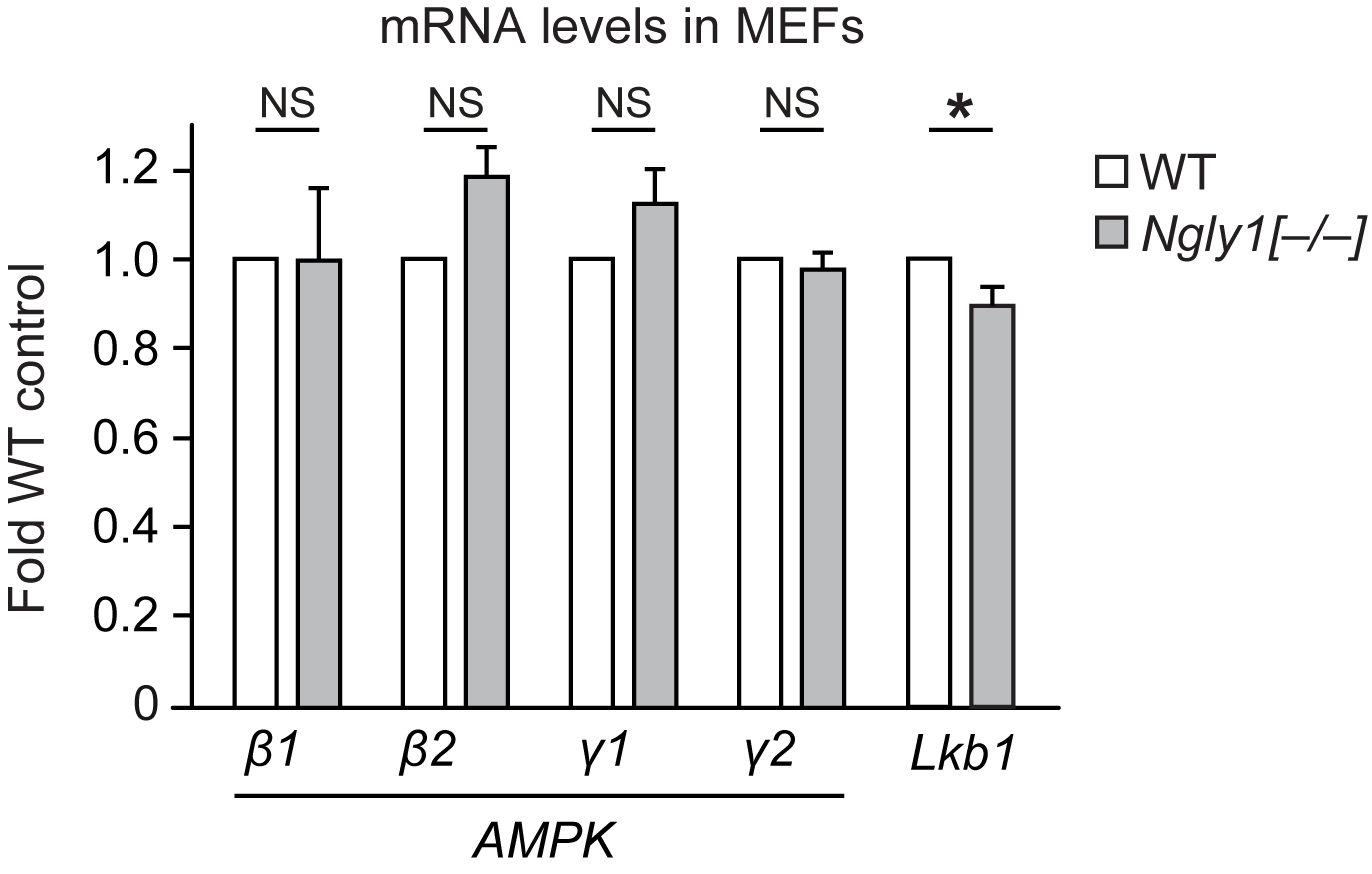

Supplement: S6 Fig — *P<0.05. NS, not significant. (TIF) [file pgen.1009258.s006.tif]

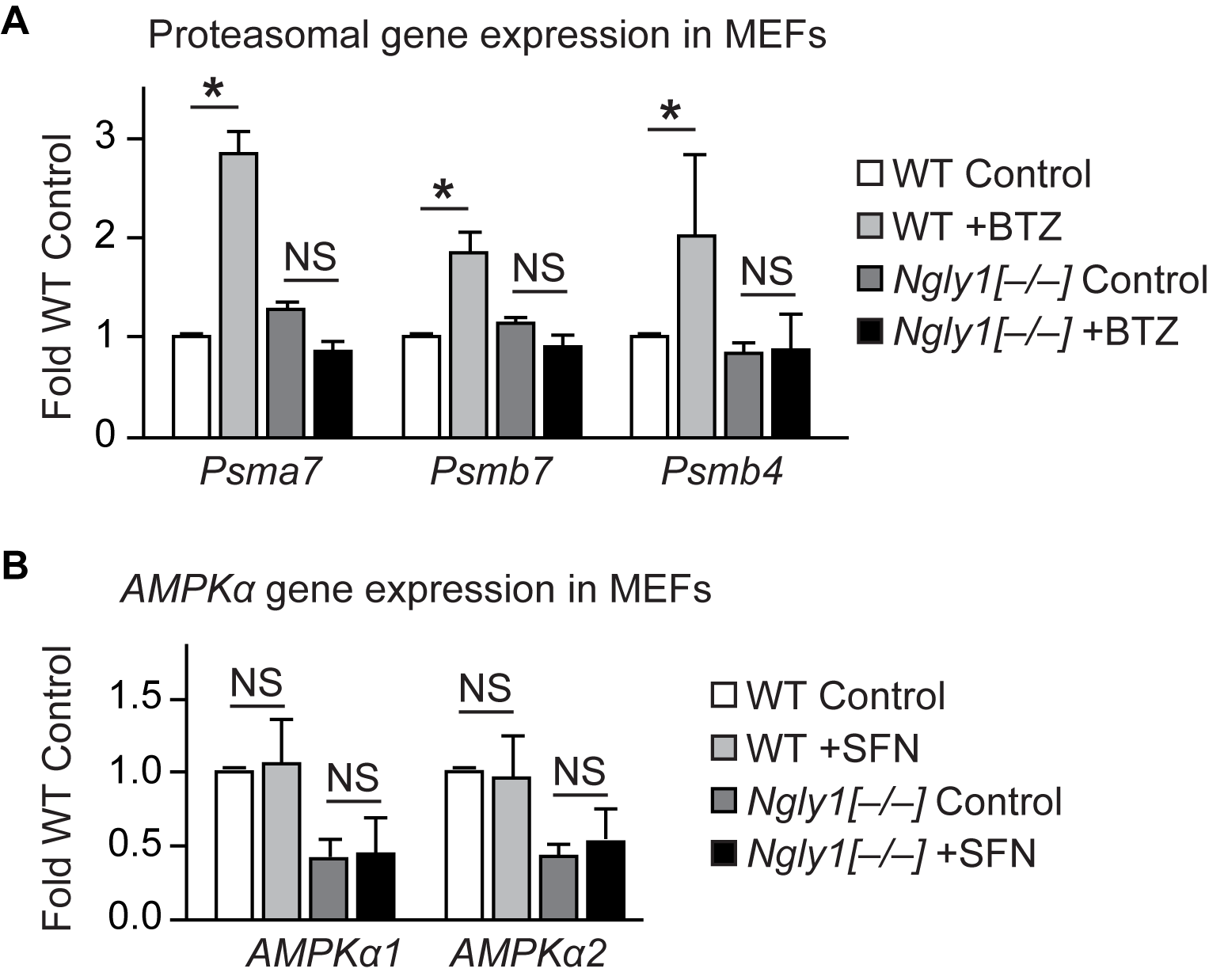

Supplement: S7 Fig — (A) qRT-PCR assays show relative mRNA levels of proteasomal (Psm) genes in control and Bortezomib (BTZ)-treated wild-type and Ngly1–/– MEFs. Note the impaired proteasome bounce-back response in Ngly1–/– MEFs, in agreement with a previous report [11]. (B) SFN treatment does not increase the level of AMPKα1 and AMPKα2 mRNA in Ngly1–/– MEFs. (TIF) [file pgen.1009258.s007.tif]

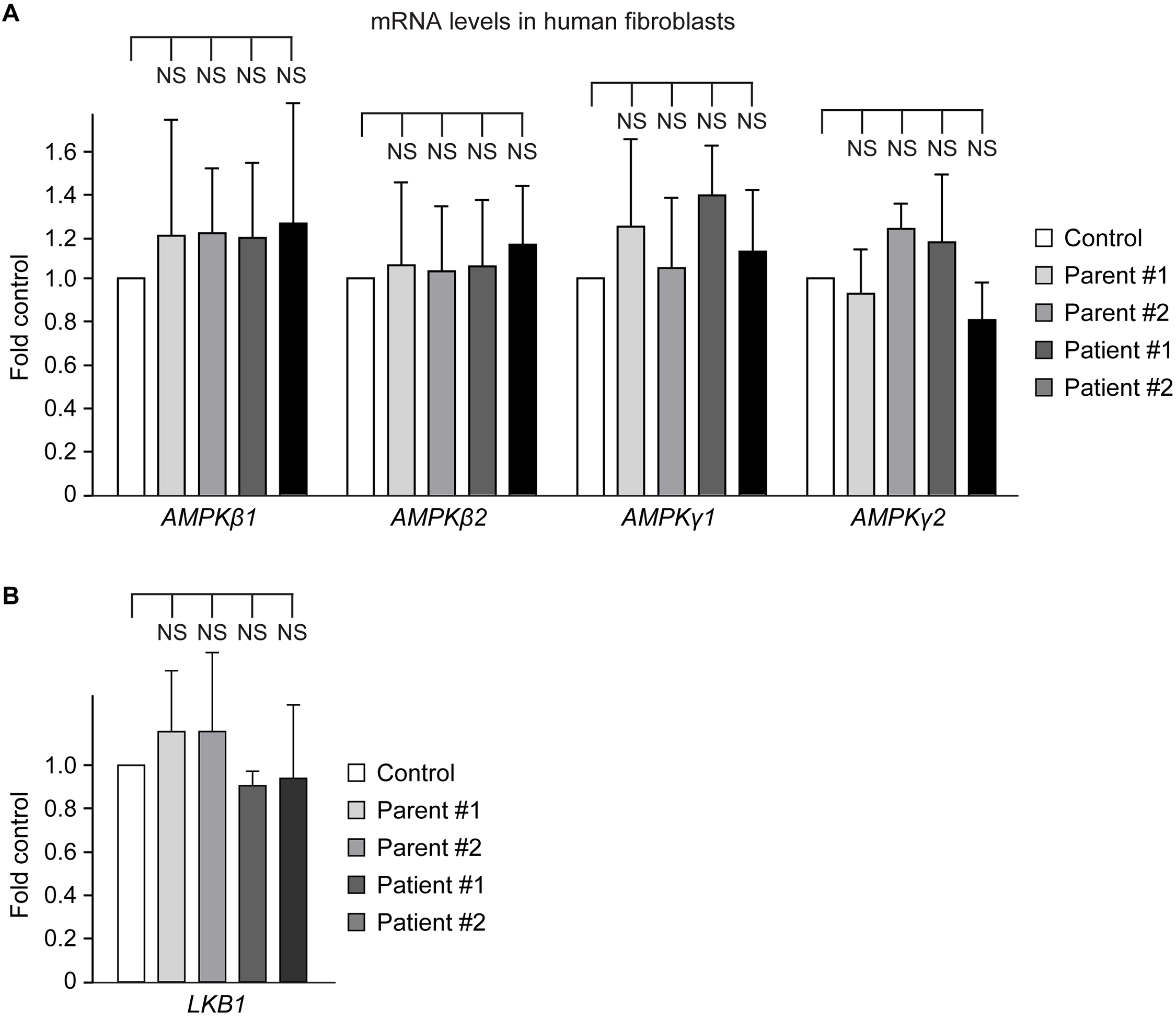

Supplement: S8 Fig — NS, not significant. (TIF) [file pgen.1009258.s008.tif]
